# Multisensory integration of speech sounds with letters vs. visual speech: only visual speech induces the mismatch negativity

**DOI:** 10.1111/ejn.13908

**Published:** 2018-04-01

**Authors:** Jeroen J. Stekelenburg, Mirjam Keetels, Jean Vroomen

**Affiliations:** ^1^ Department of Cognitive Neuropsychology Tilburg University Warandelaan 2, PO box 90153 5000 LE Tilburg the Netherlands

**Keywords:** event‐related potentials, McGurk‐MMN, text–sound integration, visual speech–sound integration

## Abstract

Numerous studies have demonstrated that the vision of lip movements can alter the perception of auditory speech syllables (McGurk effect). While there is ample evidence for integration of text and auditory speech, there are only a few studies on the orthographic equivalent of the McGurk effect. Here, we examined whether written text, like visual speech, can induce an illusory change in the perception of speech sounds on both the behavioural and neural levels. In a sound categorization task, we found that both text and visual speech changed the identity of speech sounds from an /aba/‐/ada/ continuum, but the size of this audiovisual effect was considerably smaller for text than visual speech. To examine at which level in the information processing hierarchy these multisensory interactions occur, we recorded electroencephalography in an audiovisual mismatch negativity (MMN, a component of the event‐related potential reflecting preattentive auditory change detection) paradigm in which deviant text or visual speech was used to induce an illusory change in a sequence of ambiguous sounds halfway between /aba/ and /ada/. We found that only deviant visual speech induced an MMN, but not deviant text, which induced a late P3‐like positive potential. These results demonstrate that text has much weaker effects on sound processing than visual speech does, possibly because text has different biological roots than visual speech.

## Introduction

Experienced readers automatically and effortlessly associate letters with speech sounds. This ability suggests that letters and speech sounds are not processed in isolation, but at some processing stage are combined into a coherent multisensory representation. Behavioural evidence for a coupling between letters and speech sounds comes from studies showing that degraded speech is perceived as more clearly when presented together with written text (Frost & Katz, 1989; Sohoglu *et al*., 2014). Written text can also induce lasting changes (recalibration) in the perception of ambiguous speech sounds, as is well established for visual speech (Bertelson *et al*., 2003). For example, in studies by Keetels *et al*. (2016) and Bonte *et al*. (2017), an ambiguous speech sound halfway between /aba/ and /ada/ was coupled repeatedly with text (‘aba’ or ‘ada’). In an auditory‐only post‐test following this adaptation phase, it appeared that text had shifted the interpretation of the ambiguous sound towards /aba/ or /ada/, respectively. Neuroimaging (fMRI) studies aimed at identifying the neural correlates underlying letter–sound integration, have found that the superior temporal sulcus (STS) and auditory cortex are involved in the neural binding of text and speech (van Atteveldt *et al*., 2004). A MEG study confirmed the involvement of STS in letter–sound integration (Raij *et al*., 2000). The STS is also involved in the integration of lip movements and speech (Calvert *et al*., 1999, 2000), thus suggesting that audiovisual integration of text with speech sounds and visual speech with speech sounds shares common neural circuitry.

However, at present, it is not fully understood whether written text actually changes the percept of speech as visual speech does, and if so, at what processing stage this occurs. For audiovisual speech, there is ample evidence that visual speech can alter the percept of heard speech sounds, either by complete visual capture or by creating a fusion of two phonemes, that is the McGurk effect (McGurk & MacDonald, 1976). Only a few studies, though, have examined an orthographic equivalent of the McGurk effect (Massaro *et al*., 1988; Fowler & Dekle, 1991; Massaro, 1998), while none has compared it directly with visual speech using electroencephalography (EEG). Massaro *et al*. (1988) were among the first to report that sound identification of a /ba/‐/da/ continuum was shifted by concurrently presented text. They found that listeners reported more often ‘ba’ when sounds from the middle of the continuum were combined with the text ‘ba’ instead of ‘da’. Furthermore, this text‐induced bias was substantially smaller compared to the effect of visual speech. In the same vein, Fowler & Dekle (1991) reported a marginal biasing effect of written text (‘ba’ or ‘ga’) on sound identification of a /ba/‐/ga/ continuum. Massaro (1998), though, found a more substantial bias of written text on speech sound identification. When comparing the strength of behavioural recalibration effects reported for different types of context information, it appears that visual speech induces stronger effects than written text (Keetels *et al*., 2016). Together, this thus suggests that written text evokes a rather subtle change in sound categorization that is relatively small compared to visual speech. It remains to be examined whether these text‐induced effects reflect more response‐related biases in the sense that listeners report the letter if unsure about the sound or a shift in identification that is more perceptual in nature. Here, we therefore use EEG to further characterize these findings.

In Experiment 1, we first examined whether written text does indeed evoke a change in sound identification that is relatively small when compared to visual speech. We further tried to increase the biasing effect of text by varying the stimulus onset asynchrony (SOA) at which text is presented relative to the sound. In previous studies (Massaro *et al*., 1988; Fowler & Dekle, 1991), text was always presented at the same time as the onset of the critical consonant, but this synchronous presentation may not be optimal because further research has demonstrated that sensory predictions may play a critical role in changing the percept of what is heard (Sohoglu *et al*., 2014). From that perspective, it may be better to present the text slightly ahead of the sound instead of synchronous, a procedure that we adopted with the intention to boost the text effect.

Once stimuli and SOAs were validated, we assessed the effect of text and visual speech on sound processing with EEG using the mismatch negativity (MMN). The MMN is a component of the event‐related potential (ERP) that is elicited by sounds that violate the automatic predictions of the central auditory system (Näätänen *et al*., 1978). The MMN is obtained by subtracting the ERP of frequent ‘standard’ sounds from infrequent ‘deviant’ sounds, and it appears as a negative deflection with a fronto‐central maximum peaking around 150–250 ms from the onset of the sound change. The MMN is most likely generated in the auditory cortex and presumably reflects preattentive auditory deviance detection (Näätänen *et al*., 2007). Important for the purpose of our study is that the MMN has also been used to probe the neural mechanisms underlying the integration of information from different senses, as in the case of hearing and seeing speech. For example, in a study of Saint‐Amour *et al*. (2007), the standard and the deviant sounds consisted of the same auditory sound (/ba/), but the visual parts differed, visual speech ‘ba’ for the standard and visual speech ‘va’ for the deviant. This deviant visual stimulus (seeing /va/ instead of /ba/) created the illusion of hearing a different syllable (/va/) that in turn evoked a so‐called McGurk‐MMN. Several other studies have found similar effects (Sams *et al*., 1991; Colin *et al*., 2002; Kislyuk *et al*., 2008; Stekelenburg & Vroomen, 2012), thus demonstrating that ‘auditory’ sensory memory can be modified by visual speech. To the best of our knowledge, though, no study has used this McGurk‐MMN for letter–sound combinations using this logic.

It should be acknowledged, though, that other studies have used a MMN paradigm to investigate letter–sound integration (Froyen *et al*., 2008; Andres *et al*., 2011; Mittag *et al*., 2011), but they relied on different logic than that the deviant letter would actually change the percept of the sound. For example, Froyen *et al*. (2008) compared an auditory‐only MMN − evoked by a deviant sound /o/ differing from the standard sound /a/ − with the MMN evoked by the same deviant sound /o/ among standard /a/, but now accompanied by a simultaneously presented letter ‘a’ for both standard and deviant (audiovisual condition). The MMN amplitude in this audiovisual condition was now *enhanced* compared to the auditory‐only condition, presumably because the deviant sound /o/ now not only differed from the standard sound /a/, but also differed from the letter ‘a’. This letter–sound incongruency between ‘a’ and /o/ thus further enhanced the amplitude of the MMN. Note, even though that this logic is different from the McGurk‐MMN because if the deviant sound /o/ was actually *integrated* with letter /a/, it should have diminished the perceptual difference between the standard sound /a/ and deviant sound /o/, thus evoking a *smaller* MMN amplitude, as has indeed been reported in the study by Kislyuk *et al*. (2008) in which the MMN for auditory deviant /ba/ and standard /va/ was abolished when both sounds were accompanied by visual speech ‘va’.

To increase the chance that the MMN would actually reflect a change in the *percept* of the sound by the deviant visual stimulus rather than a change in sound–letter congruency, we used as in our previous studies (Bertelson *et al*., 2003; Keetels *et al*., 2016) an ambiguous sound halfway between /aba/ and /ada/. This ambiguous sound was presented with standard or deviant text (‘aba’ and ‘ada’, respectively) or standard and deviant visual speech (/aba/ and /ada/, respectively). We expected the *perceptual change* induced by the visual stimuli to be maximal for ambiguous sounds, while at the same time the audiovisual congruence/incongruence should be about equal for standards and deviants because the sound was always halfway between text and visual speech ‘aba’ and ‘ada’. The MMN evoked by the deviant visual stimuli would then thus reflect the ‘true’ perceptual change in the ambiguous sound.

## Experiment 1: illusory behavioural change of sound by text vs. visual speech

### Method

#### Participants

Twenty students from Tilburg University participated and received course credits for their participation (17 females, 20 right‐handed, average age 20.3 years, SD 2.2, range 18–25). Participants reported normal hearing and normal or corrected‐to‐normal seeing. All participants were fluent Dutch speakers without a diagnosis of dyslexia. They were tested individually and were unaware of the purpose of the experiment. Written informed consent was obtained from each participant (in accordance with the Declaration of Helsinki). The Ethics Review Board of the School of Social and Behavioral Sciences of Tilburg University approved all experimental procedures (EC‐2016.48).

#### Stimuli

**Figure 1:**
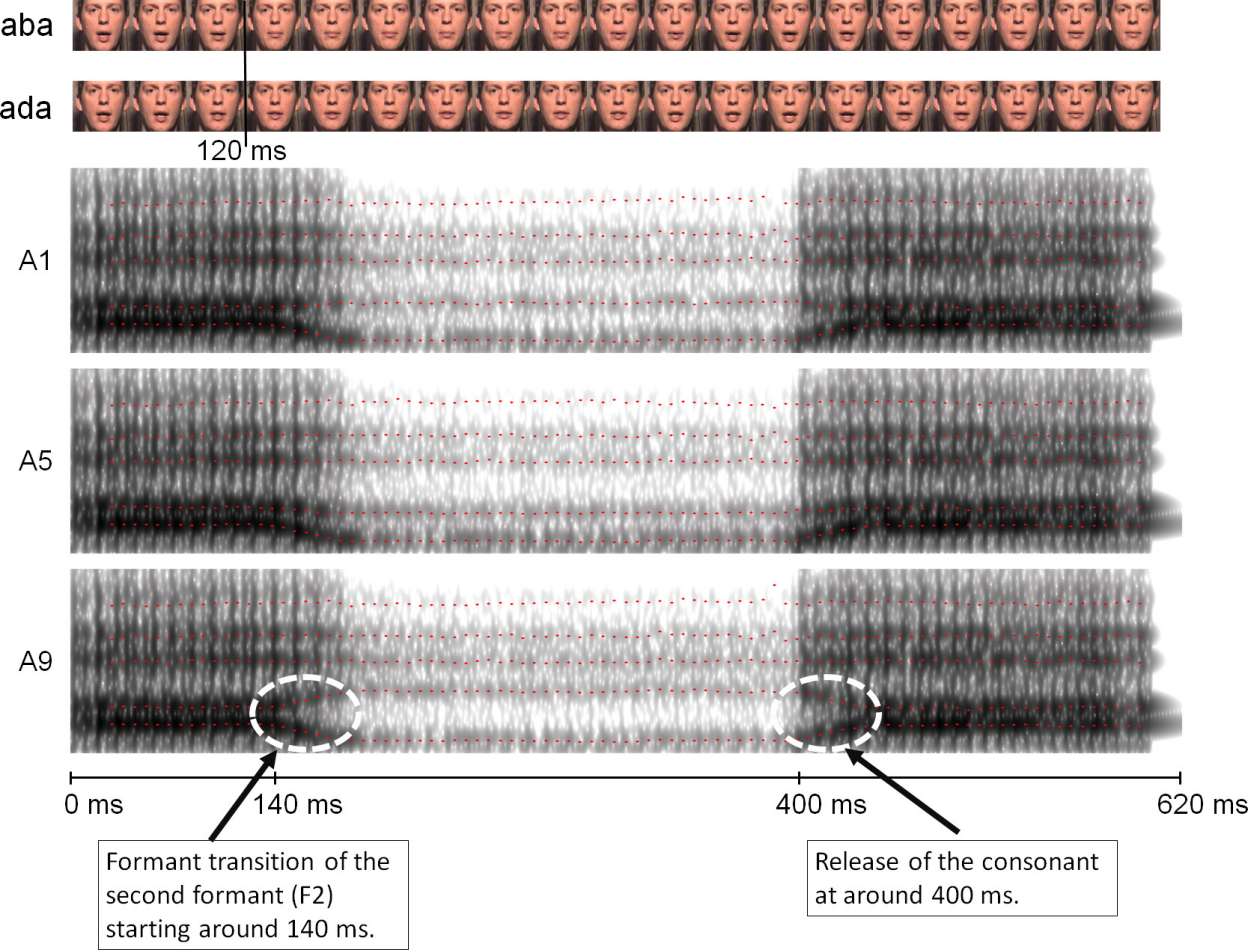
Video stills and spectrograms of auditory stimuli A1 (corresponding to /aba/), A5 (ambiguous sound halfway between /aba/ and /ada/) and A9 (/ada/). The auditory continuum was created by manipulating the formant transition of F2. The first acoustic change that signals the difference between /b/ and /d/ occurred around 140 ms after sound onset in the transition of second formant (F2) of the initial vowel. The release of the consonant was at about 400 ms after sound onset. In the video, the difference between /aba/ and /ada/ became apparent at around 120 ms. [Colour figure can be viewed at http://www.wileyonlinelibrary.com/].

The experiment took place in a dimly lit and sound‐attenuated room. Visual stimuli were presented on a 17‐inch CRT monitor at 640 × 480 pixel resolution. Sounds were presented via two loudspeakers located next to the monitor at 68 dB (A) intensity. The stimuli used in this study have been used before and are described in detail in Bertelson *et al*. (2003). In short, we used audiovisual recordings (at 25 frames/second) of a male Dutch speaker pronouncing the Dutch pseudowords /aba/ and /ada/. The visual and auditory tracks of the recordings were accustomed separately. The auditory tracks of the recordings were synthesized into a nine‐token /aba/–/ada/ continuum (henceforth A1–A9) by changing the second formant (F2) in eight steps of 39 Mel using the ‘Praat’ speech editor (Boersma & Weenink, 1999) (See Fig. 1). The average F2 consonant frequency was 1100 Hz for the /aba/ continuum endpoint and 1680 Hz for the/ada/endpoint. The duration of all sound files was 620 ms. The onset and the release of the critical consonant after onset were at approximately 140 and 400 ms, respectively (Fig. 1). Two types of visual stimuli were used, visual speech videos and text. The auditory stimuli could be presented either unimodally (A) or in combination with visual stimuli (visual speech or text). The visual speech videos consisted of the visual tracks of the audiovisual /aba/ and /ada/ recordings in which the whole face of a male actor is visible (Fig. 1). The videos had duration of 1960 ms and were displayed as a string of 49 bitmaps (including a 9 bitmap black‐to‐colour fade‐in and 9 bitmap colour‐to‐black fade‐out) in which each bitmap was displayed for 40 ms at a refresh rate of 100 Hz. For visual speech stimuli, the visual onset of the consonant was at about 120 ms after sound onset. The onset was determined as the first video frame (the fourth video frame) in which lip closure was visible after lip‐opening of the initial vowel. The image size was 9 × 6.5 deg (height × width) and was presented on a black background at the centre of the screen. The text stimuli were the pseudowords ‘aba’ and ‘ada’. The ink colour of the letter stimuli was white and these were presented on a black background in the centre of the screen in lowercase letters (5.5 × 2.5 deg). Duration of the written text was 1200 ms. The audiovisual timing of the visual speech stimuli was natural (no delay), whereas the onset of the text (aba or ada) was presented either synchronously with the initial vowel of the /a?a/ sound or 200 ms earlier, so that the timing of the text relative to the critical consonant was about 140 or 340 ms earlier, respectively.

#### Design and procedure

The task of the participants was to identify the sounds as either /aba/ or /ada/ using two dedicated buttons, while ignoring (but still watching) the visual stimulus. The sounds were presented either with or without a visual stimulus. This amounted to a total of four conditions: auditory‐only; AV speech; letter–sound synchronized to the initial vowel (so the text precedes the onset of the critical consonant by ~140 ms); and letter–sound asynchronous (the text precedes the onset of the critical consonant by ~340 ms). To avoid that participants were exposed to unnatural and highly incongruent AV combinations (like seeing visual speech /ba/ and hearing /da/ that often yield unnatural responses like /bda/), we only combined sounds A1–A5 (/aba/ side of the continuum) with visual ‘aba’ and sounds A5–A10 (/ada/ side of the continuum) with visual ‘ada’, so that only sound A5, the most ambiguous one, was combined with both visual ‘aba’ and ‘ada’. Trial order was randomized, and each unique trial was repeated 14 times.

### Results and discussion

**Figure 2:**
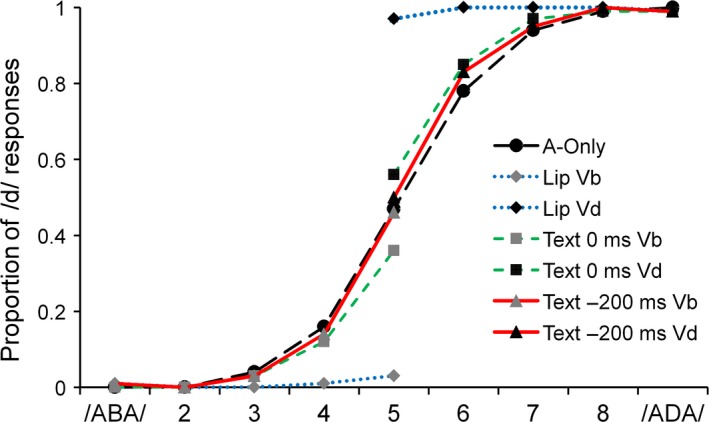
Proportion of /ada/ responses on the auditory continuum (/aba/ to /ada/) for the conditions auditory‐only (A‐only); visual speech ‘aba’ (Lip Vb) and visual speech ‘ada’ (Lip Vd) with synchronized audiovisual onset; text ‘aba’ (Text 0 ms Vb) text and ‘ada’ (Text 0 ms Vd) with synchronized audiovisual onset; text ‘aba’ (Text −200 ms Vb) and text ‘ada’ (Text −200 ms Vd) with visual onset to auditory onset lead of 200 ms. [Colour figure can be viewed at http://www.wileyonlinelibrary.com/].

Figure 2 shows the proportion of /d/ responses for each of the nine sounds for A‐only, AV speech, letter–sound synchronous and letter–sound asynchronous conditions. As is clearly visible, the psychometric curves of the AV speech condition deviated the most from the A‐only baseline, indicating that visual speech had the strongest visual effect on auditory identification. There was a small text effect for synchronous letter–sound combinations, but the text effect was virtually absent for the asynchronous condition. These observations were formally tested by two‐way repeated‐measures anovas (separate for the left side [/aba/] and the right side [/ada/] of the psychometric curve) with the within‐subject variables Condition (A‐only, AV speech, letter–sound synchronous and letter–sound asynchronous) and Token (either A1, A2, A3, A4, A5 or A5, A6, A7, A8, A9) on the log‐odds transformed proportion of /d/ responses. The log‐odds transformation was performed to meet assumptions of distribution normality (the analyses of the non‐corrected data produced similar outcomes). For the /aba/ side and /ada/ side, there was a main effect of Token (*F*_4,16_ = 16.34, *P *=* *0.00002, $\eta_{p}^{2}$ = 0.80; *F*_4,16_ = 27.47, *P *=* *0.0000005, $\eta_{p}^{2}$ = 0.87, respectively). Post hoc pairwise comparisons (Bonferroni corrected) showed that the proportion of /d/ responses differed between all tokens (all *P*‐values < 0.01), except for token pairs 1 and 2; 2 and 3; 7 and 8; and 8 and 9. Both anovas also revealed a main effect of Condition (*F*_3,17_ = 12.68, *P *=* *0.0001, $\eta_{p}^{2}$ = 0.69; *F*_3,17_ = 13.22, *P *=* *0.0001, $\eta_{p}^{2}$ = 0.70). Post hoc pairwise comparisons (Bonferroni corrected) showed that for the /aba/ side, the proportion of /d/ responses for the visual speech condition was lower (i.e. more /b/ responses) than for the other conditions (all *P*‐values < 0.001). For the /ada/ side, the proportion of /d/ responses for the visual speech condition was higher than that for the other conditions (all *P*‐values < 0.0001). For the /ada/ side, more /d/ responses were also given for the synchronous written text than those for the A‐only condition (*P *=* *0.007). The Condition × Token interactions for both /aba/ and /aba/ sides (*F*_12,8_ = 5.46, *P *=* *0.01, $\eta_{p}^{2}$ = 0.89; *F*_12,8_ = 8.16, *P *=* *0.003, $\eta_{p}^{2}$ = 0.92) were significant. Follow‐up simple effect tests on the Condition x Token interactions were conducted on the most ambiguous token (A5) because there the difference between conditions was the largest. We subtracted, per condition, the scores on the A5 sound combined with visual ‘aba’ from visual ‘ada’, resulting in an intersensory bias score. The difference score was 0.93 for AV speech, 0.20 for synchronous written text and 0.05 for the asynchronous written text, which implies that visual speech biased auditory categorization the most and asynchronous written text the least. The difference scores were entered in a repeated‐measures anova with the within‐subject variable condition (AV speech, letter–sound synchronous and letter–sound asynchronous). There was a main effect of Condition (*F*_2,18_ = 162.22, *P *=* *2.99 × 10^−12^, $\eta_{p}^{2}$ = 0.95). Post hoc (Bonferroni corrected) tests showed that each condition differed from each other (all *P*‐values < 0.01). Subsequently, one‐sample *t*‐tests on the difference scores were run to test whether the difference scores differed from zero (indicating an intersensory effect). Visual speech (difference score of 0.93) and synchronous written text (difference score of 0.20) significantly influenced auditory identification (*t*_19_ = 31.56, *P *=* *6.6 × 10^−18^, *d* = 7.08; *t*_19_ = 4.44, *P *=* *0.0003, *d* = 0.99, respectively). The difference score of the asynchronous condition (0.05) was not different from zero (*t*_19_ = 0.99, *P *=* *0.34).

Experiment 1 demonstrates that the most ambiguous speech sound from our /aba/‐/ada/ continuum was almost completely captured by concurrently presented visual speech, while written text, when presented synchronously with the onset of the vowel, had a smaller effect. These results are in line with the data of Massaro *et al*. (1988), who also reported that visual speech is more potent than text to shift the interpretation of ambiguous speech sounds. Furthermore, the fact that SOA significantly influenced the effect of written text on auditory categorization suggests the intersensory effect for written text cannot solely be explained by visual response bias.

A somewhat surprising finding was that when text was presented before the sound, it did not boost the intersensory effect, but rather diminished it when compared to the synchronized condition. This is unlike the findings of Sohoglu *et al*. (2014) who reported that the perceived clarity of degraded speech was increased when written text preceded rather than followed the degraded words. A possible explanation for the diminished effect in the 200‐ms condition is that – similar to the McGurk effect – there is a temporal window in which multisensory integration is optimal (van Wassenhove *et al*., 2007; see for a review Vroomen & Keetels, 2010). For visual speech, it is well known that there is quite a large temporal window of integration (van Wassenhove *et al*., 2007), but our behavioural results suggest that this temporal window of integration might be smaller for the integration of text and speech. A future study might assess this with more fine‐grained SOAs between text and speech.

To further examine whether the shift in sound identification by text reflects a sensory component of letter–sound integration, we investigated the neural correlates of this effect using a paradigm intended to evoke an MMN. Before conducting the audiovisual MMN experiments, we first ran an auditory‐only control MMN experiment (Experiment 2) to validate that the difference between the consonants in /aba/(token A1) and /ada/ (token A9) would elicit an MMN. This is a prerequisite for the elicitation of a McGurk‐MMN, because if no auditory‐only MMN is elicited by an actual difference between the consonants, no McGurk‐MMN is expected either. Furthermore, the auditory‐evoked MMN also served as a reference to estimate the time at which an illusory sound change induced by either visual speech or text information would penetrate the auditory system.

In Experiment 3, we tested whether deviant *visual speech* information would induce a McGurk‐MMN, while in Experiment 4, we used deviant *text*. To capitalize on that the MMN would reflect a change in sound percept *per se* rather than a change in audiovisual congruency, we used the most ambiguous speech sounds (A5, henceforward denoted as /A?/) as the auditory component for the standard and deviant audiovisual combinations. The visual component for the standard was ‘aba’, and for the deviant, it was ‘ada’. Of note, despite that a McGurk‐MMN with visual speech has been reported in several studies (Sams *et al*., 1991; Colin *et al*., 2002; Saint‐Amour *et al*., 2007; Kislyuk *et al*., 2008; Stekelenburg & Vroomen, 2012), it has yet to be established whether it can be induced with ambiguous sounds for which audiovisual (in)congruency is, arguably, more balanced in standard and deviant trials. Indeed, in previous studies, there was a potential confound of AV congruency because the standard was always AV congruent and the deviant was AV incongruent. In the current study, though, both standard and deviant are about equally (i.e. slightly) AV incongruent. We expected that visual speech would capture the identity of the speech sound so that the standard was ‘heard’ as /aba/ and the deviant as /ada/. This illusory change in sound percept was expected to evoke a McGurk‐MMN. A separate visual‐only (V‐only) condition served as control to rule out that the AV‐MMN was based on the visual difference between standard and deviant (a visual MMN) and to correct the AV‐MMN accordingly by subtracting the V‐only (deviant – standard) difference wave from the AV (deviant – standard) difference wave (Saint‐Amour *et al*., 2007; Stekelenburg & Vroomen, 2012).

## Experiment 2: auditory‐induced MMN

### Participants

We tested 22 new healthy participants (18 women, mean age 19.5 years, SD 1.1, range 18–22).

### Stimuli and procedure

The auditory stimuli were A1 (/aba/) and A9 (/ada/) of Experiment 1, representing the standard and deviant, respectively. There were 600 standards (80%) and 150 deviants (20%) administered across three blocks. Trial order was randomized with the restriction that at least two standards preceded each deviant. The interstimulus interval (ISI), measured from sound to sound onset, was 1880 ms. Participants listened passively to the sounds with their eyes open.

### EEG recording and analysis

The EEG was recorded at a sample rate of 512 Hz from 32 locations using active Ag‐AgCl electrodes (BioSemi, Amsterdam, The Netherlands) mounted in an elastic cap and two mastoid electrodes. The electrodes were placed according to the international 10–20 system. Horizontal and vertical eye movements were recorded using electrodes at the outer canthus of each eye and above and below the right eye, respectively. Two additional electrodes served as reference (Common Mode Sense active electrode) and ground (Driven Right Leg passive electrode). EEG was referenced offline to an average of left and right mastoids and band‐pass‐filtered (0.5–30 Hz, 24 dB/octave). The 50 Hz interference was removed by a 50‐Hz notch filter. The raw data were segmented into epochs of 1100 ms, including a 100‐ms prestimulus baseline. ERPs were time‐locked to sound onset of the initial vowel. After EOG correction (by applying the Gratton *et al*. (1983) algorithm in which ocular artefacts were corrected by subtracting the EOG channels, multiplied by a channel‐dependent correction factor from the EEG channels), epochs with an amplitude change exceeding ± 120 μV at any EEG channel were rejected (average rejection rate for standard and deviant was 12.7 and 13.2%, respectively). The epochs were averaged separately for standards and deviants. Individual difference waves were computed by subtracting the averaged ERP of the standard from the averaged ERP of the deviant. The difference wave was tested against prestimulus baseline levels by point‐by‐point two‐tailed *t*‐tests at each electrode in a 1‐ to 1000‐ms window. Using a procedure to minimize type I errors (Guthrie & Buchwald, 1991), the difference wave was considered significant when at least 12 consecutive points (i.e. ~23 ms) were significantly different from zero.

### Results and discussion

**Figure 3:**
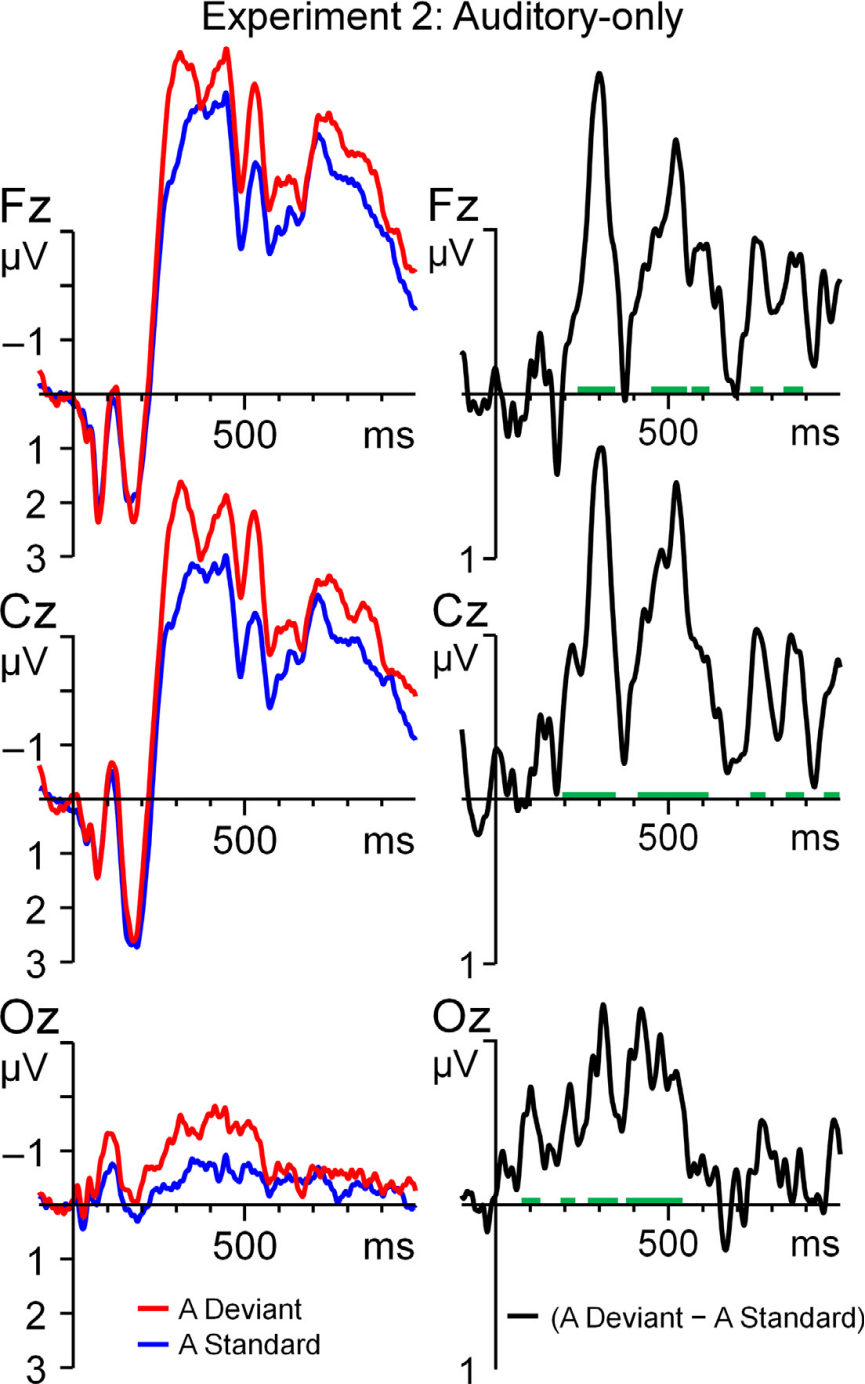
Grand average ERPs for the A‐only experiment (Experiment 2), time‐locked to sound onset. The left panel shows the standard and deviant and the right panel the deviant–standard difference wave. The green trace indicates epochs where the difference wave significantly (*P* < 0.05) deviates from zero. [Colour figure can be viewed at http://www.wileyonlinelibrary.com/].

The auditory /ada/ deviant evoked a clear MMN as shown in Figs 3 (ERP) and 4 (running *t*‐tests). The running *t*‐test revealed multiple phases in the MMN at ~90 to 150 ms with an occipito‐parietal scalp distribution, ~180 to 350 ms, and ~420 to 650 ms, both with a central distribution. The mean activity was calculated per phase and separately entered in a repeated‐measures anova with the within‐subject variable Electrode (PO3, POZ, PO4, O1, Oz, O2 for the 90–150 ms phase and Fz, FC1, FC2, C3, Cz, C4, CP1, CP2, Pz for the 180–350 ms and 420–650 ms phases). For all three phases, the mean activity was more negative than zero (*F*_1,21_ = 7.42, *P *=* *0.01, $\eta_{p}^{2}$ = 0.26; *F*_1,21_ = 56.11, *P *=* *2.3 × 10^−7^, $\eta_{p}^{2}$ = 0.73; *F*_1,21_ = 15.56, *P *=* *0.0007, $\eta_{p}^{2}$ = 0.43, respectively), independently of Electrode (all *P*‐values > 0.08). Considering the early onset and the posterior topography of the first phase, it is unlikely that it was elicited by differences in the initial vowel because the spectrograms and sound intensities of the standard and deviant were identical until about 140 ms after sound onset. We therefore doubt that the first phase is directly linked to acoustical changes in the deviant, but instead reflect noise in the data that is insufficiently cancelled out by averaging. The timing of the second and third phases suggests that these phases probably reflect auditory differences related to the closure and the release of the consonant, respectively. In Experiment 3, we then tested whether these MMN components could be elicited by an illusory change in the consonant – induced by visual speech stimuli – rather than an acoustic change.

**Figure 4:**
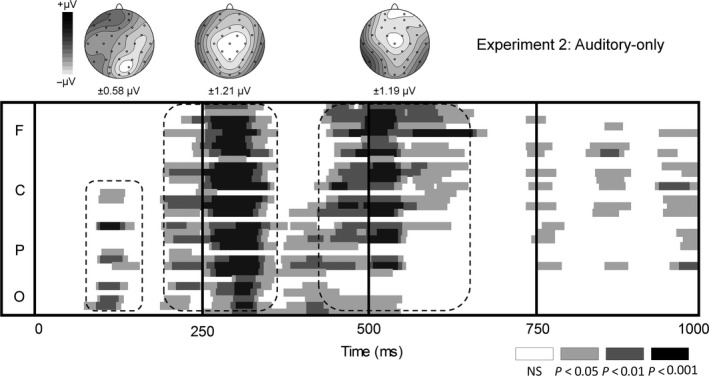
Time course of the deviant − standard difference wave for the A‐only control experiment (Experiment 2) using pointwise *t‐*tests against prestimulus baseline at every electrode (frontal [F], central [C], parietal [P] and occipital [O]) and topographies (in μV) for the different phases in the MMN.

## Experiment 3: visual speech‐induced MMN

### Participants

We tested 21 new healthy participants (9 women, mean age 19.8 years, SD 1.6, range 18–23).

### Stimuli and procedure

The visual speech stimuli were identical to the ones used in Experiment 1. The magnitude of the MMN is sensitive to the interstimulus interval (ISI), with larger MMNs for shorter ISIs (Näätänen *et al*., 2007). To improve the conditions to obtain a robust MMN, we therefore stripped a few frames from the video in which there was no essential movement to keep the ISI as short as possible. There were two different conditions comprising V‐only and AV stimulus presentations. Each condition contained 600 standards and 150 deviants (20%), administered across three blocks per condition. In the AV condition, the standard was auditory /A?/ combined with visual ‘aba’ (denoted as A?Vb), while the deviant was auditory /A?/ combined with visual ‘ada’ (A?Vd). For the V‐only condition, the standard was the visual speech stimulus ‘aba’ and the deviant was visual speech ‘ada’ (Vb and Vd, respectively). Trial order was randomized with the restriction that at least two standards preceded each deviant. The V‐only and AV blocks alternated with block‐order counterbalanced across participants. The interstimulus interval, measured from sound onset in the AV blocks or the corresponding time stamp in the V blocks, was 1880 ms (identical to Experiment 2). Participants were not actively engaged in processing the identity of the sound, but to ensure that they were watching the screen during stimulus presentation they had to detect, by key press, catch trials that consisted of the occasional occurrence of a white dot on the upper lip (10% catch trials of the total number of trials were restricted to the standard) for both AV and V conditions.

### EEG recording and analysis

Electroencephalography recording was identical to that described in Experiment 2. The average rejection rate after artefact rejection for V‐only standard and deviant was 9.8 and 9.2%, respectively. The rejection rate for both AV standard and deviant was 11.5%. ERPs were time‐locked to sound onset of the initial vowel in the AV condition or to the corresponding time stamp in the V‐only condition. The epochs of the non‐catch trials were averaged for standards and deviants, separately for the V‐only and AV blocks. Individual difference waves per modality were computed by subtracting the averaged ERP of the standard from the averaged ERP of the deviant. The difference wave in the AV condition may be composed of overlapping components pertaining to the illusory change in the sound as well as the change in text. To suppress ERP activity evoked by the visual change, the difference waveform (deviant – standard) of the V‐only condition was subtracted from the difference waveform (deviant – standard) of the AV condition. This AV‐V difference wave represents the EEG activity evoked by the illusory change in the sound in its purest form, thus without contribution of the visual component (Saint‐Amour *et al*., 2007; Stekelenburg & Vroomen, 2012).

The analysis started with the exploration of the spatiotemporal properties of the AV‐V difference wave. To track the time course of the intersensory effect on the MMN, we conducted point‐by‐point *t‐*tests on the AV‐V difference wave. The AV‐V difference wave was tested against prestimulus baseline levels by point‐by‐point two‐tailed *t‐*tests at each electrode in a 1‐ to 1000‐ms window. As in Experiment 2, we used a procedure to minimize type I errors (Guthrie & Buchwald, 1991) in which the AV‐V difference wave was considered significant when at least 12 consecutive points (i.e. ~23 ms) were significantly different from zero.

### Results and discussion

Participants detected 96% (SD 5%) of the catch trials, indicating that participants complied with instructions and were watching the screen.

**Figure 5:**
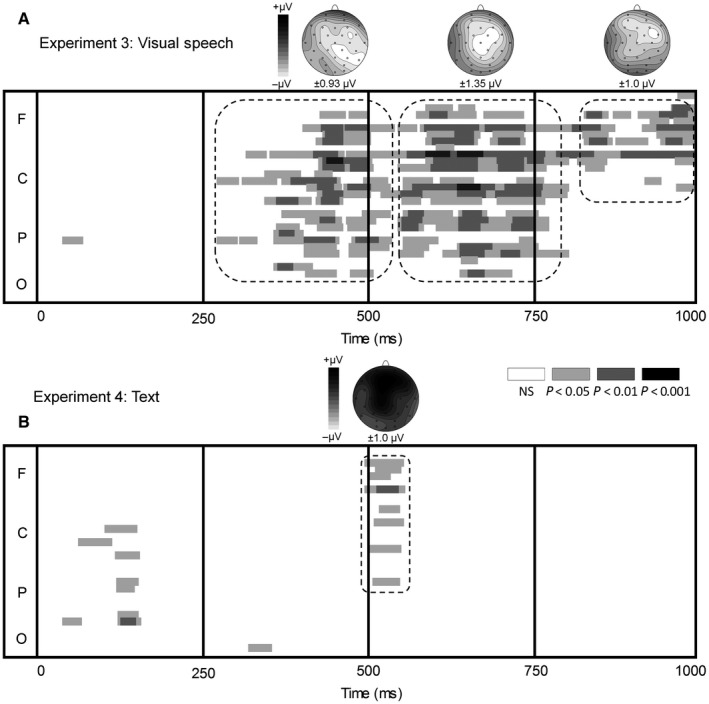
Time course of the AV (AV deviant – AV standard) − V (V deviant – V standard) difference wave using pointwise *t‐*tests against prestimulus baseline at every electrode (frontal [F], central [C], parietal [P] and occipital [O]) and topographies (in μV) for the visual speech experiment (a, Experiment 3) and text experiment (b, Experiment 4).

**Figure 6:**
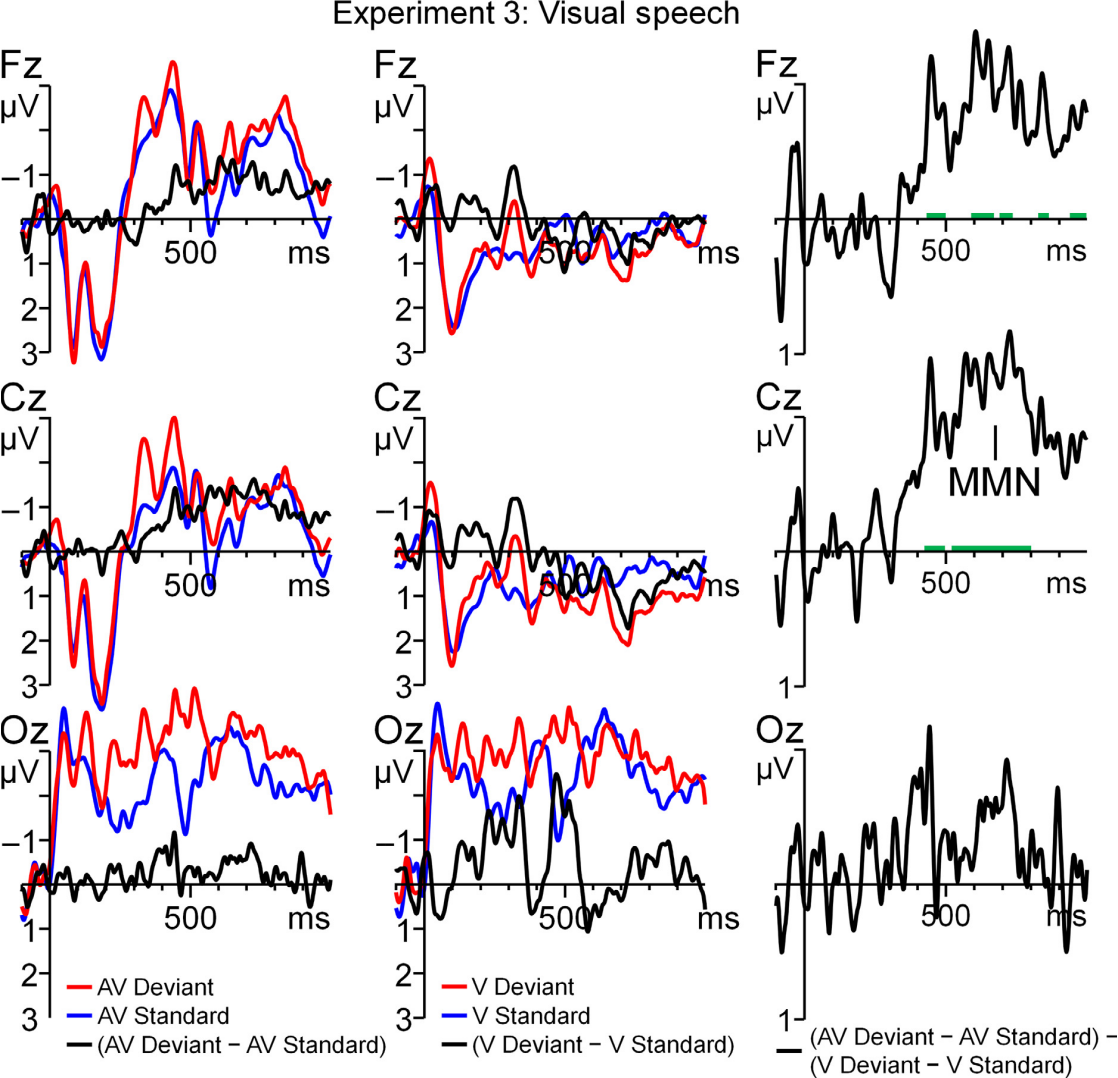
Grand average ERPs, time‐locked to auditory onset for the visual speech experiment. The left and middle panels denote the standard, deviant and the deviant – standard difference wave for the V‐only and AV conditions, respectively. In the right panel, the AV (AV deviant – AV standard) − V (V deviant – V standard) difference wave is displayed. The green trace indicates epochs where the AV − V difference wave significantly (*P* < 0.05) deviates from zero. [Colour figure can be viewed at http://www.wileyonlinelibrary.com/].

As shown in Figs 5a and 6, the AV‐V difference wave was consistently more negative than prestimulus baseline across a prolonged window starting at about 280 ms after the initial vowel onset (i.e. 140 ms after the onset of the ambiguous consonant). Visual inspection of Fig. 5a further indicates that there were three clusters (280–540 ms; 550–770 ms; 810–1000 ms) with distinct topographies. The mean activity for those clusters was calculated per cluster and separately entered in a repeated‐measures anova with the within‐subject variable Electrode (Fz, FC1, FC2, C3, Cz, C4, CP1 CP2, Pz for the 280‐ to 540‐ms and 550‐ to 770‐ms windows and AF3, AF4, F3, Fz, F4, FC1, FC2 for the 810‐ to 1000‐ms window). For all three windows, the mean activity was more negative than zero (*F*_1,20_ = 12.32, *P *=* *0.002, $\eta_{p}^{2}$ = 0.38; *F*_1,20_ = 14.06, *P *=* *0.001, $\eta_{p}^{2}$ = 0.41; *F*_1,20_ = 11.85, *P *=* *0.003, $\eta_{p}^{2}$ = 0.37, respectively), independently of Electrode (all *F*‐values < 1).

Experiment 3 shows that deviant visual speech that is combined with an ambiguous speech sound elicits a robust McGurk‐MMN. This is in line with other studies on the McGurk‐MMN that used clear and non‐ambiguous speech sounds (Sams *et al*., 1991; Colin *et al*., 2002; Saint‐Amour *et al*., 2007; Stekelenburg & Vroomen, 2012). The novel finding is that the current results exclude the possibility that the McGurk‐MMN is induced by a change in audiovisual congruence of the deviant. Rather, it is more likely that an illusory change in sound identity evokes a McGurk‐MMN. A future study might further investigate the contribution of AV congruency to the McGurk‐MMN by manipulating stimulus probability. An 80% standard 20% deviant condition may be compared to a 50/50% condition. No MMN is to be expected for the 50/50% condition if the McGurk‐MMN is solely the result of an illusory change in sound identity. However, if an MMN‐like response would be found for the 50/50% condition, this can be ascribed to AV congruency.

The McGurk‐MMN in our study consisted of three phases. This is in line with Experiment 2 in which we also found multiple phases in the MMN, even though these acoustically evoked MMN started earlier (~180 ms). As MMN latency is longer with decreasing stimulus deviation (Näätänen *et al*., 2007), a possible explanation for the relative late onset of the McGurk‐MMN is that the visually induced bias resulted in a smaller perceptual difference between standard and deviant when compared to the auditory‐only condition.

## Experiment 4: text‐induced MMN

In Experiment 4, we tested whether similar results could be obtained when text instead of visual speech was used to induce an illusory change of the sound. We anticipated that the text‐induced MMN might be smaller on the basis of Experiment 1, and to ensure that potential null results could not be explained by undersampling, we increased our sample size from 21 participants (in Experiment 3) to 36 participants (26 women, mean age 19.8 years, SD 1.9, range 18–28). The experimental design (including the ratio of standards to deviants and ISI), measurements and analyses were identical to Experiment 3, except that visual speech was replaced by synchronized text for the duration of 1200 ms. The text was synchronized with sound onset (so ~140 ms before the onset of the critical consonant). During catch trials, the text changed from lower‐ to uppercase. The average rejection rate after artefact rejection for V‐only standard and deviant was 17.3 and 16.5%, respectively. The rejection rate for AV standard and deviant was 13.5 and 12.8%, respectively.

### Results and discussion

Participants detected 98% (SD 4%) of the catch trials.

**Figure 7:**
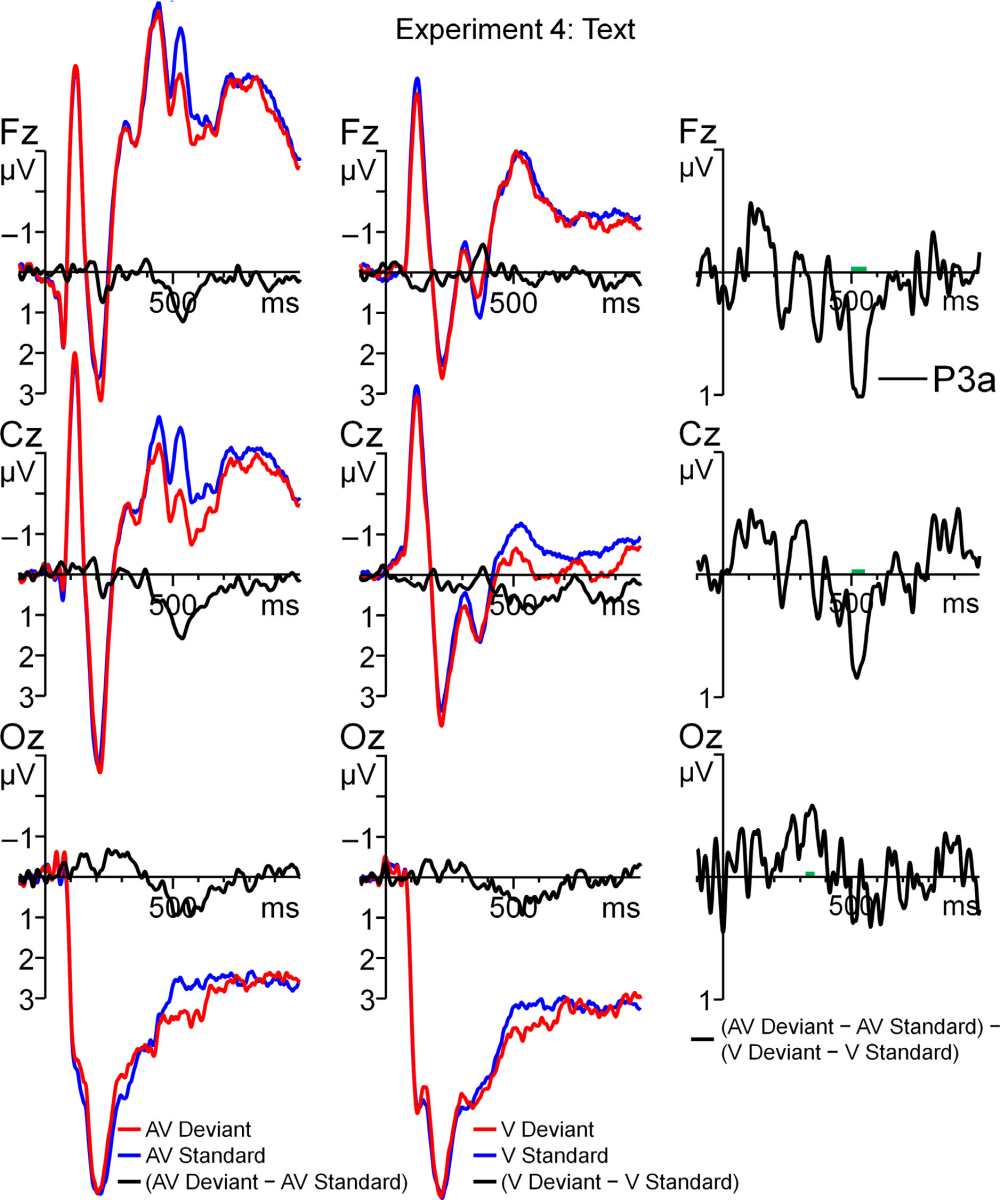
Grand average ERPs, time‐locked to auditory onset for the written text experiment (Experiment 4). The left and middle panels denote the standard, deviant and the deviant – standard difference wave for the V‐only and AV conditions, respectively. In the right panel, the AV (AV deviant – AV standard) − V (V deviant – V standard) difference wave is displayed. The green trace indicates epochs where the AV − V difference wave significantly (*P* < 0.05) deviates from zero. [Colour figure can be viewed at http://www.wileyonlinelibrary.com/].

Figure 7 shows the ERPs for the standard and deviant (only non‐catch trials were included) with their corresponding difference waves for the AV and V conditions. The most prominent deflection of the difference waves for both AV and V was a fronto‐central P3 at 450–600 ms (reminiscent of the P3a). The analysis of the running *t‐*tests showed two clusters that deviated from prestimulus baseline activity (Fig. 5b). In the window of 120–150 ms, the AV‐V difference wave shows a negative deflection at the left centro‐parietal electrodes. In the window of 500–550 ms, the AV‐V difference wave was positive at the mid‐frontal electrodes. We tested the mean activity of the two temporal windows in a repeated‐measures anova with the within‐subject variable Electrode (C3, CP1, CP5 and P3 for the 120‐ to 150‐ms window; AF3, AF4, F3, Fz, F4, FC1 and FC2 for the 500‐ to 550‐ms window). For both early and late windows, mean activity differed from zero (*F*_1,35_ = 10.42, *P *=* *0.003, $\eta_{p}^{2}$ = 0.23; *F*_1,35_ = 7.61, *P *<* *0.009, $\eta_{p}^{2}$ = 0.18, respectively). There were no main effects of Electrode (all *P*‐values > 0.24).

The results of Experiment 4 show that − after correction for visual deviancy − the AV deviant was more negative than the standard at 120–150 ms after sound onset. It is, however, difficult to interpret this early effect as a result of a change in the perception in the consonant because it occurs before (~140 ms) the auditory transition from the vowel to the consonant. Admittedly, we have at present no clear explanation for this early negativity. It might reflect the V‐only MMN to differences in text (‘ada’ vs. ‘aba’) that for unknown reasons was larger in the AV condition. Most importantly, we found no MMN, but a positive deflection with a frontal distribution that is indicative of a P3a. The timing of the P3a (500–550 ms after sound onset) suggests that it was time‐locked to the release of the consonant and that it matched the timing of the late MMN in A‐only experiment (Experiment 2), which we linked to perceived deviancy of the release of the consonant.

It is apparent that the audiovisual difference wave evoked by written text is qualitatively different from the one by visual speech because visual speech evoked a negative deflection (i.e. MMN) instead of a P3a. Differences in activity may originate from multiple sources, which will be discussed in general discussion.

## General Discussion

This study examined, at the behavioural and neural levels, whether written text – just like visual speech – evokes a change in sound identity. The results of Experiment 1 demonstrate that written text, when presented synchronous with the onset of the sound, could bias the percept of a sound, but to a significantly lesser degree than visual speech. Experiments 3 and 4 examined these intersensory effects with a McGurk‐MMN paradigm. Although Experiment 4 found neural activity associated with a change of written text, this was qualitatively different from Experiment 3 that used visual speech. The most striking difference is that the deviancy‐related activity showed a positive deflection (P3a) for written text, whereas there was a negative deflection (MMN) for visual speech. The MMN for visual speech is in line with other studies on the McGurk‐MMN (Sams *et al*., 1991; Colin *et al*., 2002; Saint‐Amour *et al*., 2007; Stekelenburg & Vroomen, 2012) and suggests that this effect is indeed based on the fact that visual speech changes the percept of the consonant at a sensory level.

A relevant question, then, is why there is a no text‐induced McGurk‐MMN. It is unlikely to be due to statistical undersampling because we used substantially more participants in Experiment 4 (text) than Experiment 3 (visual speech, 36 vs. 21, respectively). An alternative explanation for the null result is that audiovisual integration of letters and sounds might take place after the stage of auditory deviancy detection. This would be in line with an MEG study on letter–sound integration reporting late AV congruency effects after 450 ms in STS (Raij *et al*., 2000). However, several studies report earlier letter–sound congruency effects. Hu *et al*. (2012), for example, found interactions at 180–210 ms. Furthermore, Herdman *et al*. (2006) found letter–sound congruency effects that were localized in the auditory cortex in a 0‐ to 250‐ms time window. The study of Herdman *et al*. (2006) suggests that graphemes facilitate early sensory processing in the auditory cortex. Early letter–sound interaction is also advocated by MMN studies using clear speech tokens (Froyen *et al*., 2008; Andres *et al*., 2011; Mittag *et al*., 2011), which implies that letter–sound interactions do occur prior to the MMN. The question then is why no MMN was elicited in our study? A factor that might account for differences between letter–sound integration in the behavioural task (Experiment 1) and the MMN task (Experiment 4) might relate to differences in attention paid to the letters. In Experiment 1, speech sounds had to be rated while letters were to be ignored, whereas in Experiment 4, speech sounds and letters were both task‐irrelevant. What these two experiments have in common is that the tasks themselves did not require AV integration and that the letters were always task‐irrelevant. It seems therefore doubtful that differences in attention paid to letters would explain differences between the behavioural and neural data and why this putative effect of attention would solely occur for letters, but not visual speech. A future study, though, might explore whether letter–sound integration at the neural level can be boosted by requiring participants to relate the speech sounds and text to each other (cf. Raij *et al*., 2000). The most plausible explanation for the absence of a text‐induced MMN is that the intersensory bias is simply too small to evoke the MMN as it is known that the magnitude of the MMN is dependent on the perceived difference between the standard and the deviant (Pakarinen *et al*., 2007). Our behavioural results support this argument because the intersensory bias by text was only about 20% in magnitude (which is relatively small compared to visual speech of 93%). The neural generators underlying the text‐MMN may therefore not have been sensitive enough to respond to the rather subtle influence of text on sound identity.

How do our results relate to other studies that did report an effect of written text on MMN while using clear speech (Froyen *et al*., 2008; Andres *et al*., 2011; Mittag *et al*., 2011)? As already mentioned, these studies relied on a different strategy to examine whether a visual change in an audiovisual stimulus induces an MMN. For example, Froyen *et al*. (2008) found that the audiovisual MMN was larger than the auditory‐only MMN if both the standard /a/ and deviant /o/ were synchronized by a congruent letter to the standard sound (/a/). From the Froyen *et al*. (2008) study, it may be concluded that letter–sound integration occurs at a relatively early, preattentive stage in stimulus processing as they found that the auditory‐only (non‐illusory) MMN induced by a deviancy in speech sounds is affected by concurrently presented written text. However, because in the AV condition of Froyen *et al*. (2008), the standard comprised congruent AV stimuli whereas the deviant comprised incongruent AV stimuli, it might be the case that the deviant ERP was composed of incongruency‐related activity as such. Therefore, this study cannot distinguish between perceptual effects of visual letters on auditory sensory memory vs. effects evoked by a change in congruency. In an extension of the study of Froyen *et al*. (2008), Mittag *et al*. (2011) recorded the MMN to speech sound changes when they were presented with either letters congruent with the auditory standard or nonsense visual stimuli. The MMN was larger when auditory deviants were paired with written syllables compared to nonsense visual stimuli. Unfortunately, also in this study, audiovisual congruency effects could account for the increased amplitude of the MMN. In a study by Andres *et al*. (2011) that was specifically aimed at disentangling congruency and sensory memory effects, participants listened to auditory standards (/o/) and deviants (/i/) while watching visual letters that were occasionally synchronized to the auditory stimuli. Half of the synchronized AV trials were AV congruent, and half were incongruent. Although both congruent and incongruent AV trials elicited a significant MMN, the MMN for congruent AV pairings was larger, indicative of an effect of written text on the auditory MMN. The difference between the incongruent and congruent MMN, however, was found at a parietal electrode (Pz) and not at the frontal sites where both the MMN of the congruent and incongruent AV stimuli reached their topographical maximum. Manipulating AV congruency may have activated an additional neural generator in one of both conditions that projected to the posterior electrodes on the scalp. It might therefore be questioned whether the difference between the incongruent and congruent MMN reflects a difference in the change detection process in the auditory cortex or rather an AV congruency effect. We conjecture that the results of the above‐discussed MMN studies could also be explained by letter–sound congruency and do not provide conclusive evidence that written text actually changes preattentive auditory deviancy detection.

An issue that needs to be addressed is why a P3a‐like response (with its maximum at Fz) was elicited in Experiment 4 in the absence of the MMN, because the P3a is generally preceded by the MMN (Escera & Corral, 2007). We can conceive of several possibilities; first, it might be that P3a is somehow an artefact of the AV minus V subtraction as detection of the visual deviant might have elicited the MMN in the V‐only condition but not, or less so, in the AV condition resulting in a net positive deflection in the AV‐V difference wave. This account of the P3a, however, is refuted by the data showing no P3a in the V‐only condition at electrode Fz and a clear P3a at Fz in the AV condition. The joint presentation of the sound and the visual stimuli thus seems to be conditional upon the evocation of the P3a. If the P3a is not an artefact of the applied experimental paradigm, could it be that written text influenced sound identity not at the level of where an MMN is generated, but at a processing stage that reflects the P3a? This would be problematic considering that it is generally acknowledged that the functional significance of the P3a (in a passive oddball design) is an involuntary shift of attention towards the deviant, which is a tightly coupled to the MMN (Escera & Corral, 2007). In other words, an attention shift (P3a) is thought to only occur if auditory deviancy is detected (MMN). However, there are also reports of a decoupling of the MMN and P3a (Horvath *et al*., 2008) on the basis of which they conjectured that the P3a is evoked by significant events in a general sense. In the current case, the significant event could be the detection of a mismatch between the visual and auditory stimuli. We speculate that due to repetition of the standard stimulus, participants learned to associate the ambiguous sound with the text ‘aba’. In that context, the occasional deviant (with the visual ‘ada’) was perceived as incongruent relative to the learned AV association of the standard. We therefore conjecture that the P3a probably reflects a higher‐level event detection process not related to an actual shift of the auditory percept.

To conclude, the current study demonstrated that written text has a considerably smaller effect on speech sound identification than visual speech. At the neural level, visual speech, but not written text, induced the McGurk‐MMN. Our results stress the importance of delineating AV congruency and sensory memory effects. The effect of written text on auditory MMN in studies using clear speech (Froyen *et al*., 2008; Mittag *et al*., 2011) can be attributed to AV congruency affecting the ERP, without any modulation of auditory speech identity. However, without any contribution of AV congruency effects to the MMN, we found no effect of written text on the MMN. This implies that a conflict between speech and written text is processed within 200 ms, but written text does not change the perceived auditory identity at the level of the MMN. The relatively subtle effect of written text on auditory phoneme categorization – intended to induce illusory auditory differences between standard and deviant − proved to be insufficient to exceed the threshold for preattentive auditory deviancy detection. The absence of the McGurk‐MMN for text stimuli does not necessarily dismiss the possibility that letter–sound integration takes place at the perceptual stage in stimulus processing, but rather that the behavioural task in the current study seemed to be more sensitive than the MMN approach to elucidate the effect of written text on the auditory speech percept. The finding of stronger intersensory coupling for visual speech stimuli is not surprising considering the strong biological constraints between perception and production that may already be present at birth, whereas for written text the coupling of letters and speech sounds is arbitrary and requires explicit learning in a later stage of life.

## Conflict of interest

The authors report no conflict of interests.

## Data accessibility

Stimuli and data are available upon request.

## Author contributions

JS and MK designed the experiments. MK and JS analysed Experiment 1. JS analysed Experiments 2–4. JS drafted the manuscript. JS, MK and JV edited the manuscript. All authors approved the final version.
