# Supplementary material for: Multisensory integration of speech sounds with letters vs. visual speech: only visual speech induces the mismatch negativity
Source: Eur J Neurosci. 2018 Apr 1;47(9):1135–45. doi: 10.1111/ejn.13908 (PMC5969231; doi:10.1111/ejn.13908)
Supplement: Supplementary file 1 [file EJN-47-1135-s001.pdf]

EJN European Journal of Neuroscience FENS Federation of European Neuroscience Societies

Multisensory integration of speech sounds with letters versus visual speech: Only visual speech induces the mismatch negativity

Jeroen J. Stekelenburg, Mirjam Keetels & Jean Vroomen

Review timeline:

Submission date:	14 September 2017
Editorial Decision:	31 October 2017
Revision received:	05 December 2017
Editorial Decision:	15 January 2018
Revision received:	12 February 2018
Accepted:	06 March 2018

Editor: John Foxe

1st Editorial Decision 31 October 2017

Dear Dr. Stekelenburg,

Your manuscript has now been thoroughly reviewed by three expert reviewers who have provided detailed commentaries on the paper. Both the reviewers and the editors appreciate the important topic and interesting approach. We have somewhat divergent opinions of the readiness of the manuscript, however, and so a fairly substantial revision is called for before we can proceed. As you will read below, Reviewer #1 is very satisfied with the study as reported and lists only relatively minor modifications that will require your attention. The other two reviewers point to more serious issues, however. Reviewer #2 calls for more balance in your set-up of the study, which is very concentrated on the text aspect of the experiment. She also provides suggestions for ways to make the text flow better, and asks for clarifications about your methods and analysis strategies. For example, how did you choose electrodes to enter into your analysis, and how does one conceptualize the dissociation of MMN from basic AV integration effects? There are suggestions for ways to clarify the illustrations also.

Reviewer #3 is the most critical. He points out that the lack of an MMN in Experiment 4 is not consonant with the fact that you find a statistically significant behavioral effect in Experiment 1 for the text stimuli, and that your results are not in agreement with some of the prior literature on the subject. He makes the case that the paradigm in Experiment 4 may be the culprit here since it did not require the same level of engagement on the part of the participants with the bisensory aspect of the stimulus materials (unlike Expt. 1). You will need to deal with this potential issue in your revision, and might want to consider recording an additional dataset to directly address the issue of attentional engagement. This, we will leave to your discretion.

Please also attend to the following issues in your revision:
1. Your abstract could do a better job of motivating the study.
2. Please be sure to provide the age range of your participants.
3. Footnotes should be removed or relocated to the main text.
4. Your P values are reported as inequalities – please report them in full per EJN guidelines
5. Better resolution figures will be needed
6. In a similar vein, the figures are rather small and hard to read at present – please revise.
7. We wonder if the second instance of “audiovisual” in your title might not read better as “multisensory”?

If you are able to respond fully to the points raised, we would be pleased to receive a revision of your paper within 12 weeks.

Thank you for submitting your work to EJN.

Kind regards,

John Foxe & Paul Bolam
co-Editors in Chief, EJN

EJN European Journal of Neuroscience FENS Federation of European Neuroscience Societies

Reviews:

Reviewer: 1 (Kaisa Tiippana, University of Helsinki, Finland)

Comments to the Author
The manuscript “Audiovisual integration of speech sounds with letters versus lipread speech: Only lipread speech induces audiovisual mismatch negativity (MMN)” by Stekelenburg, Keetels and Vroomen reports a well-conducted study, which is appropriate for EJN. The main finding is that the MMN is elicited by visual speech, but not by text, which reflects the perceptual findings of strong visual influence on auditory speech perception by the former, but not the latter stimuli. The manuscript is well written with appropriate referencing, the design and findings are clear, and the study provides a novel contribution. I find the manuscript acceptable already as it is, and only have some minor comments and suggestions.

Minor points:
• I don’t like the word “lipreading” in the context of this manuscript since to me it means speechreading, i.e. visual-only speech, and in this study the main message is about how visual speech influences auditory speech processing. I’d prefer replacing “lipreading” by “visual speech”.
• Perhaps a brief explanation of the MMN would be useful in the abstract.
• “sync” could be spelled out as “synchrony/ous”.
• Sometimes the present tense is used in the Results instead of the past tense.
• The figures with waveforms could include the prestimulus baseline, and the legend could state that horizontally Fz, Cz, Oz are shown. The final figures will hopefully be more easily legible.
• p. 7: The title of Experiment 1 could include the word “behavioral”.
• p. 8: “For lipread stimuli, the visual onset of the consonant was at about 120 ms after sound onset.” How was this determined?
• p. 9: “In order to avoid that participants were exposed to unnatural and highly incongruent AV combinations (like seeing lipread /ba/ and hearing /da/) that often yield unnatural responses like /bda/”. Why call the so-called combination stimuli and responses “unnatural”?
• p. 11: The finding on asynchronous text could be discussed further. The authors hypothesized a larger effect when the text leads because of its predictive value. Why did they instead find the opposite, i.e. no effect with leading text and a weak effect with synchronous text? Their finding is in line with the MMN findings of Froyen & al. (2008) and Mittag & al. (2011) who showed a reduced MMN with asynchronous text.
• p. 21, 23: The discussion on in/congruency could be clarified. Importantly, what is the in/congruency effect, and how exactly is it a confounding factor? For example: “due to the applied experimental design of the Froyen et al. (2008) study, the deviant ERP may be composed of incongruency-related activity that is evoked regardless of the standard. Therefore, study cannot distinguish between effects of visual letters on auditory sensory memory and AV incongruency effects as were found in the deviant.” In addition, what is meant by “letters homologous to the auditory standard”?

Reviewer: 2 (Sarah Haigh, University of Pittsburgh, USA)

Comments to the Author
This study focused on comparing two types of audio-visual deviant where the visual cue was either a video of lipreading or text. The authors found a large MMN in the lipreading condition, and a smaller P3 to the text condition. This paper provides an interesting method for exploring the multisensory mechanisms involved in language processing. I have a few comments to add.
Introduction: most of the introduction is focused on text involvement in language perception. Is there not much literature focused on lipreading?
Experiment 1:
1) It would be helpful to highlight that this is a behavioural task, including in the subheading.
2) The way that the statistics are written does not help the reader’s understanding of what is significant from what. Can the authors add some context?
3) The results of the different SOA between audio and text suggest that SOA hurts the potential effect text has on perception. Does not this suggest that any SOA (even the remaining small SOA) could still be biasing the results?
4) The discussion skips around from discussing the results from Experiment 1 to the rationale behind Experiment 3 and 4, then to Experiment 2. Could the authors clarify this section?
Experiment 2:
1) More EOG correction information is needed.
2) Please add statistics to the results section.
3) There is little discussion about the timing of the MMN. In the introduction, the MMN is described as being around 150-250ms after deviant onset, but all the MMNs listed in the paper are much later. Can the authors comment on this?
Experiment 3:

1) The videos were shortened. Can the authors elaborate?
2) When describing the EEG analysis, was the V-only deviant waveform subtracted from the AV deviant waveform? This step of the analysis is unclear.
3) The method of subtracting V-only from AV seems a little strange. The difference waveform would include deviant activity AND multisensory information. From the subtraction waveforms in Figure 6, it seems that the difference between V-only standard and V-only deviant was small and the same was true for the AV condition. Therefore, the MMN seems to be due to larger negativity in the AV condition, that was present in both standard and deviant waveforms. Can the authors provide a better rationale for their method, when the A-only condition used the typical deviant-standard method? Might the 'early MMN' be due to the effect of A+V?
4) Why do the electrodes included in the statistics differ depending on the time-points used? How were these electrodes selected?
Experiment 4:
1) Did the text still differ in SOA from the audio?
2) The analytical and statistical comments from those listed for Experiment 3 are also relevant here
General Discussion:
1) The presence of a P3 in the AV-text condition does not constitute a null result. Please clarify.
2) There is a small problem of generalising across these studies as each experiment contained new participants. It is possible that the AV-text participants generated smaller ERPs overall, which could account for these results. Was there any overlap in participant population?
Figures:
1) Figure 1 – what is the important difference we are supposed to see here?
2) Figure 2 – the legend needs to be explained
3) The waveforms are very small – can these be enlarged?
4) Can the significant parts of the waveforms be highlighted?

Reviewer: 3 (Tommi Raij, Northwestern University, USA)

Comments to the Author
In the manuscript "Audiovisual integration of speech sounds with letters versus lipread speech: Only lipread speech induces audiovisual mismatch negativity (MMN)" Sketelenburg and colleagues report behavioral and ERP findings for audiovisual stimuli, specifically for (a) lipread speech (McGurk) stimuli and (b) text stimuli.

The goal is to investigate if MMN/McGurk type responses could be found, not only for lipreading speech stimuli, but also for AV text stimuli. This is an interesting question, as lipreading speech (visual input from faces while listening to speech) is learned throughout life starting at a very early age, whereas letters of the alphabet are culture-dependent concepts where the picture-sound pairings are arbitrary and there is less exercise that starts at a later age. Given that letters are less rehearsed and more artificial, one might expect that if there is a MMN, it would be weaker.

The changes in visual stimuli could not only change auditory processing and percepts, but also evoke visual MMNs. Due to volume conduction, these responses may extend to the frontal sensors where the auditory / MMN components were recorded. Here, to mitigate this potential artifact, the authors subtracted the responses to V-only stimuli from the responses to AV stimuli. This strategy is a strength.

In Experiment 1, the authors find that the perceptual effects are weaker for text stimuli than for lipreading speech (McGurk) stimuli. This suggests that the MMN might be weaker for letters than for lipreading speech. However, the behavioral effects were statistically significant for both types of stimuli. Whenever there is a behavioral effect, there should be a neuronal mechanisms underlying this. The question is, can this be detected with EEG ERPs.

In Experiment 2, the manuscript replicates previously known auditory MMN findings using their own auditory-only stimuli (Figure 3). As expected, the rare deviants evoke stronger responses than the frequent standard stimuli. This is a useful control.

In Experiment 3, the manuscript replicates previous ERP findings using lipreading AV speech and the McGurk effect (Figure 4). This also is a useful control.

In Experiment 4, the manuscript uses text stimuli combined with perceptually ambiguous speech auditory stimuli (Figure 7). This is the main experiment. The key finding is that there is no difference between responses to standard and deviant stimuli. Thus, Experiment 4 is in apparent conflict with Experiment 1. Experiment 4 results also seem to contradict several previous publications on this subject (see below).

The topic is well suited for European Journal of Neuroscience. The authors are experts in this field, and for the most part, the experiments are well designed, analyzed. The key findings and their interpretation are controversial, which makes them interesting.

There are also some concerns, described in more detail below, that decrease the strength of conclusions that can be drawn. As a result, I cannot recommend this manuscript for publication in its present form. A revision could change the situation.

MAIN CRITIQUE

The short title of the manuscript is "No McGurk MMN text-sound integration". However, this conclusion is possibly an artifact caused by behavioral task selection. Specifically, the tasks given to the subjects were not matched between the Experiment 1 (behavioral) and Experiment 4 (text AV). In Experiment 1, the subjects were given a task that required them to integrate the auditory and visual stimuli. In contrast, in Experiment 4, the task was to attend to the visual stimuli and push button for occasional target stimuli (uppercase), which did not require integrating the auditory and visual text. As the authors correctly note on p. 20, "the magnitude of the MMN is dependent on the perceived

difference between the standard and the deviant (Pakarinen et al., 2007)". Thus, it is to be expected that Experiment 1 found an effect for text, but Experiment 4 did not. Therefore, it would be possible to argue that the present manuscript does not have ERP data to investigate what actually happens during AV integration of letters.

Lipreading speech (McGurk-type) designs do not necessarily involve a task requiring AV integration, as the percepts are changed merely by attending to the stimuli. Thus, there is no need for an overt task that requires AV integration. It should also be noted that the magnitude of the perceptual McGurk illusion seems to be correlated with the related brain responses (Benoit et al., 2010).

Along the same lines, several previous publications have reported MMN-type responses using behavioral tasks that do not require audiovisual integration of text and speech (e.g., Froyen et al., 2008; Andres et al., 2011; Mittag et al., 2011). Such findings would seem to suggest that AV integration of text is also automatic. This makes the present negative result particularly novel and interesting.

To clarify the role of the tasks, it might be worthwhile to collect new data similar to Experiment 4 but to employ a task that requires the subjects to integrate the visual and auditory text. However, it would also be possible to interpret the present results in the light of a recently published report suggesting that audiovisual attention boosts ERPs associated with letter-sound integration (Mittag et al., 2013). To clarify, in my opinion the present results are already interesting enough to merit publication, without addition of new data.

To clarify the above, it would be useful to add in Discussion a brief review of the role of tasks in previous literature and how these may have influenced the results. The currently offered speculations on why the ERP results disagree with previous studies (Discussion pp. 20-22) seem somewhat of a stretch, but interesting and novel nevertheless.

MINOR POINTS

It was unclear if the deviant probability (apparently 20%) was the same across all ERP studies. Please clarify.

For Experiment 3 the manuscript states the following:
"The novel finding is that the current results exclude the possibility that the McGurk-MMN is induced by a change in audiovisual congruence of the deviant. Rather, it is more likely that an illusory change in sound identity evokes a McGurk-MMN." It could be interesting to relate this conclusion to the results in (Sams et al., 1991) where it would appear that stimulus probability, and not AV congruency, was the factor driving the McGurk-type difference response. Related to this, in Discussion, would it make sense to separate probability (effective ISI, separately for standards and deviants) vs. AV congruency effects?

Judging by the pre-stimulus baselines in Figures 3, 6, and 7, the data in Experiments 3-4 are quite noisy, more so than for Experiment 2. Can you suggest any reason why this might be? What were the numbers of accepted trials? For ERPs, please report the numbers of accepted trials for each experiment, and within each experiment, for each averaging class.

Figure 5 shows scalp topography results for both Experiment 3 (lipread speech) and Experiment 4 (text stimuli). The figure legend lists these as for the "AV-V difference wave". Is this merely the AV-V subtraction, and if so, for standards or deviants? Or was there first the subtraction AV-V, and then the subtraction deviant-standard? The caption would benefit from clarification.

For Experiment 4 it was unclear if the target ("catch") trials were averaged separately or together with the non-target trials. These should be averaged separately. Please clarify.

Discussing the results of Andres et al (2011) on pp. 21-22 the authors write:
"It may, however, be questioned whether the neural generators underlying the change detection process in the auditory cortex were actually affected by written text because the difference between the incongruent and congruent MMN was found at a parietal electrode (Pz) and not at the frontal sites where both the MMN of the congruent and incongruent AV stimuli had their topographical maximum. It is therefore uncertain whether the intersensory effect at Pz can actually be traced back to a difference in strength of the neural generators underling the MMN."
The MMN has generators not only in the temporal auditory cortices but also in the frontal and parietal cortex (e.g., (Alho, 1995; Rinne et al., 2000; Opitz et al., 2002; Molholm et al., 2005; Restuccia et al., 2005)). Thus, particularly since the present manuscript does not use source localization, and because the spatial relationship between scalp EEG and brain generators is complex, this conclusion seems premature. Moreover, if I am reading Figure 4 correctly, it seems that the MMN topography in present Experiment 2 was maximal at Cz/Pz, not at Fz.

In the above excerpt from Discussion, note the typo ("underling").

In future analyses, would it be possible to take the between-subjects differences in the degree of perceptual effects taken into account ? See for example (Benoit et al., 2010).

In Discussion, it could be useful to separate interaction (A+V-AV) vs MMN paradigms when discussing early vs. late congruency effects. Perhaps it could also be useful to note that cross-modal activations and interactions between auditory and visual cortices in the human brain seem ubiquitous, as the supratemporal auditory cortex may be activated by visual stimuli even without any auditory associations (checkerboards) starting at about 75 ms after stimulus onset, followed by audiovisual interactions starting at about 80 ms (Raij et al., 2010).

No source analysis (localizing the cortical generators of the scalp EEG responses) was used. The spatial relations between scalp EEG signals and their intracranial generators is very complex, especially for sulcal sources where the scalp maxima can be very far from the generators. Thus, it is unclear where in the brain the observed ERPs were generated, which hampers comparisons against studies that have used source analysis techniques. The sensor-space scalp topography maps are shown, but these are not very informative. I do not think that in the present study the lack of source analysis is a problem severe enough to prevent publication, but including it would have increased the appeal.

Figure 6 legend should state that the results are for Experiment 3 (for example, see Figure 3 legend). Figure 7 legend should state that the results are for Experiment 4 (for example, see Figure 3 legend).

References

Alho K (1995) Cerebral generators of mismatch negativity (MMN) and its magnetic counterpart (MMNm) elicited by sound changes. Ear Hear 16:38-51.

Andres A, Cardy J, Joanisse M (2011) Congruency of auditory sounds and visual letters modulates mismatch negativity and P300 event-related potentials. Int J Psychophysiol 79:137-146.

Benoit M, Raij T, Lin F, Jääskeläinen I, Stufflebeam S (2010) Primary and multisensory cortical activity is correlated with audiovisual percepts. Hum Brain Mapp 31:526-538.

Froyen D, Van Atteveldt N, Bonte M, Blomert L (2008) Cross-modal enhancement of the MMN to speech-sounds indicates early and automatic integration of letters and speech-sounds. Neurosci Lett 430:23-28.

Mittag M, Takegata R, Kujala T (2011) The effects of visual material and temporal synchrony on the processing of letters and speech sounds. Exp Brain Res 211:287-298.

Mittag M, Alho K, Takegata R, Makkonen T, Kujala T (2013) Audiovisual attention boosts letter- speech sound integration. Psychophysiology 50:1034-1044.

Molholm S, Martinez A, Ritter W, Javitt D, Foxe J (2005) The neural circuitry of pre-attentive auditory change-detection: an fMRI study of pitch and duration mismatch negativity generators. Cereb Cortex 15:545-551.

Opitz B, Rinne T, Mecklinger A, von Cramon D, Schröger E (2002) Differential contribution of frontal and temporal cortices to auditory change detection: fMRI and ERP results. Neuroimage 15:167-174.

Raij T, Ahveninen J, Lin F, Witzel T, Jääskeläinen I, Letham B, Israeli E, Sahyoun C, Vasios C, Stufflebeam S, Hämäläinen M, Belliveau J (2010) Onset timing of cross-sensory activations and multisensory interactions in auditory and visual sensory cortices. Eur J Neurosci 31:1772-1782.

Restuccia D, Della Marca G, Marra C, Rubino M, Valeriani M (2005) Attentional load of the primary task influences the frontal but not the temporal generators of mismatch negativity. Brain Res Cogn Brain Res 25:891-899.

Rinne T, Alho K, Ilmoniemi R, Virtanen J, Näätänen R (2000) Separate time behaviors of the temporal and frontal mismatch negativity sources. Neuroimage 12:14-19.

Sams M, Aulanko R, Hämäläinen M, Hari R, Lounasmaa O, Lu S, Simola J (1991) Seeing speech: visual information from lip movements modifies activity in the human auditory cortex. Neuroscience Letters 127:141-145.

Authors' Response 05 December 2017

Response to reviewers

Comments from the editor

1. Your abstract could do a better job of motivating the study.
   Response: We changed the abstract accordingly.

2. Please be sure to provide the age range of your participants.
   Response: We now do.

3. Footnotes should be removed or relocated to the main text.
   Response: We have relocated the footnote to the main text.

4. Your P values are reported as inequalities – please report them in full per EJN guidelines
   Response: We now do.

5. Better resolution figures will be needed
   Response: We did a complete overhaul on the figures.

6. In a similar vein, the figures are rather small and hard to read at present – please revise.
   Response: We did a complete overhaul on the figures.

7. We wonder if the second instance of "audiovisual" in your title might not read better as "multisensory"?
   Response: We think we even have a better title: "Multisensory integration of speech sounds with letters versus visual speech: Only visual speech induces the McGurk mismatch negativity (MMN)"

Reviewer 1

•I don't like the word "lipreading" in the context of this manuscript since to me it means speechreading, i.e. visual-only speech, and in this study the main message is about how visual speech influences auditory speech processing. I'd prefer replacing "lipreading" by "visual speech".
   Response: We replaced lipread by visual speech.

•Perhaps a brief explanation of the MMN would be useful in the abstract.
   Response: We added a short explanation: "To examine at which level in the information processing hierarchy these multisensory interactions occur, we recorded electroencephalography (EEG) in an audiovisual mismatch negativity (MMN, a component of the event-related potential [ERP] reflecting pre-attentive auditory change detection) paradigm in which deviant text or visual speech was used to induce an illusory change in a sequence of ambiguous sounds halfway between /aba/ and /ada/. "

•"sync" could be spelled out as "synchrony/ous".
   Response: we replaced sync by synchronous.

•Sometimes the present tense is used in the Results instead of the past tense.

Response: We now only use past tense, when appropriate.

•The figures with waveforms could include the prestimulus baseline, and the legend could state that horizontally Fz, Cz, Oz are shown. The final figures will hopefully be more easily legible.

Response: We completely revised the figures of the waveforms. It should be noted though that in the original figures the prestimulus baseline was already visible.

•p. 7: The title of Experiment 1 could include the word “behavioral”.

Response: We now included the word behavioral: EXPERIMENT 1: ILLUSORY BEHAVIORAL CHANGE OF SOUND BY TEXT VERSUS VISUAL SPEECH

•p. 8: “For lipread stimuli, the visual onset of the consonant was at about 120 ms after sound onset.” How was this determined?

Response: It was at the onset of the fourth video frame (of 40 ms each) after sound onset. We included this information in the revised manuscript: “The onset was determined as the first video frame (the fourth video frame) in which lip-closure was visible after lip-opening of the initial vowel. ”

•p. 9: “In order to avoid that participants were exposed to unnatural and highly incongruent AV combinations (like seeing lipread /ba/ and hearing /da/) that often yield unnatural responses like /bda/”. Why call the so-called combination stimuli and responses “unnatural”?

Response: In daily conversations the visual and auditory speech are congruent (i.e. natural). AV speech becomes unnatural when visual and auditory speech clearly contradict (in the sense that that this does not occur in real world situations). It is thus unnatural that a speaker produces a visual ‘ada’ and auditory ‘aba’ at the same time.

•p. 11: The finding on asynchronous text could be discussed further. The authors hypothesized a larger effect when the text leads because of its predictive value. Why did they instead find the opposite, i.e. no effect with leading text and a weak effect with synchronous text? Their finding is in line with the MMN findings of Froyen & al. (2008) and Mittag & al. (2011) who showed a reduced MMN with asynchronous text.

Response: We now discussed this finding in more depth: “A somewhat surprising finding was that, when text was presented before the sound, it did not boost the intersensory effect, but rather diminished it when compared to the synchronized condition. This is unlike the findings of (Sohoglu et al., 2014) who reported that the perceived clarity of degraded speech was increased when written text preceded rather than followed the degraded words. A possible explanation for the diminished effect in the 200 ms condition is that – similar to the McGurk effect – there is a temporal window in which multisensory integration is optimal (van Wassenhove et al., 2007; see for a review Vroomen & Keetels, 2010). For visual speech, it is well-known that there is quite a large temporal window of integration (van Wassenhove et al., 2007), but our behavioral results suggest that this temporal window of integration might be smaller for the integration of text and speech. A future study might assess this with more fine-grained SOAs between text and speech.”

•p. 21, 23: The discussion on in/congruency could be clarified. Importantly, what is the in/congruency effect, and how exactly is it a confounding factor? For example: “due to the applied experimental design of the Froyen et al. (2008) study, the deviant ERP may be composed of incongruency-related activity that is evoked regardless of the standard. Therefore, study cannot distinguish between effects of visual letters on auditory sensory memory and AV incongruency effects as were found in the deviant.” In addition, what is meant by “letters homologous to the auditory standard”?

Response: Two factors can contribute to the evocation of the audiovisual MMN: 1) AV congruency effects in the ERP, elicited by just combining incongruent auditory and visual (speech) stimuli. 2) Actual changes in the auditory percept by visual speech (McGurk effect) triggering pre-attentive auditory change detection. In the Froyen et al. (2008) study the auditory MMN (standard /a/; deviant /o/) was compared to the audiovisual MMN (both auditory standard /a/ and deviant /o/ were paired to visual ‘a’). Note that the standard consists of congruent AV stimuli and the deviant of incongruent AV stimuli. The AV MMN was larger than the A-only MMN. We have no way of telling whether that effect is due to factor 1 (congruency effect in the deviant) or factor 2 (auditory change

detection). To make this more clear we have rewritten this specific section: “However, because in the AV condition of Froyen et al. (2008), the standard comprised congruent AV stimuli whereas the deviant comprised *in*congruent AV stimuli, it might be the case that the deviant ERP was composed of incongruency-related activity as such. Therefore, this study cannot distinguish between perceptual effects of visual letters on auditory sensory memory versus effects evoked by a change in congruency.

-What is meant by “letters homologous to the auditory standard”?

Response: We changed homologous to “congruent with”.

Reviewer 2

Introduction: most of the introduction is focused on text involvement in language perception. Is there not much literature focused on lipreading?

Response: Compared to text, the literature on lipreading is indeed more extensive. Please bear in mind that the major focus (and the novelty of the paper) is on text. We feel that the relevant literature on lipreading was discussed, at least to such extent that it was adequate to justify the validity of our research question.

Experiment 1:
1) It would be helpful to highlight that this is a behavioural task, including in the subheading.

Response: We now included the word behavioral: EXPERIMENT 1: ILLUSORY BEHAVIORAL CHANGE OF SOUND BY TEXT VERSUS VISUAL SPEECH

2) The way that the statistics are written does not help the reader’s understanding of what is significant from what. Can the authors add some context?

Response: We added post-hoc test for the main effects. Token main effect: “Post-hoc pair-wise comparisons (Bonferroni corrected) showed the proportion /d/-responses differed between all tokens (all p-values < .01), except for token pairs 1 and 2; 2 and 3; 7 and 8; 8 and 9.”
Condition main effect: “Post-hoc pair-wise comparisons (Bonferroni corrected) showed that for the /aba/ side the proportion /d/-responses for the visual speech condition was lower (i. e. more /b/-responses) than for the other conditions (all p-values < .001). For the /ada/ side, the proportion /d/-responses for the visual speech condition was higher than for the other conditions (all p-values < .0001). For the /ada/ side, more /d/-responses were also given for the synchronous written text than for the A-only condition (P = .007).”

3) The results of the different SOA between audio and text suggest that SOA hurts the potential effect text has on perception. Does not this suggest that any SOA (even the remaining small SOA) could still be biasing the results?

Response: We now discuss this is issue in the discussion of Experiment 1: “A somewhat surprising finding was that, when text was presented before the sound, it did not boost the intersensory effect, but rather diminished it when compared to the synchronized condition. This is unlike the findings of (Sohoglu et al., 2014) who reported that the perceived clarity of degraded speech was increased when written text preceded rather than followed the degraded words. A possible explanation for the diminished effect in the 200 ms condition is that – similar to the McGurk effect – there is a temporal window in which multisensory integration is optimal (van Wassenhove et al., 2007; see for a review Vroomen & Keetels, 2010). For visual speech, it is well-known that there is quite a large temporal window of integration (van Wassenhove et al., 2007), but our behavioral results suggest that this temporal window of integration might be smaller for the integration of text and speech. A future study might assess this with more fine-grained SOAs between text and speech.”

4) The discussion skips around from discussing the results from Experiment 1 to the rationale behind Experiment 3 and 4, then to Experiment 2. Could the authors clarify this section?

Response: In the revised manuscript we elaborate more on the rationale for Experiment 2: ”Before conducting the audiovisual MMN experiments, we first ran an auditory-only control MMN experiment (Experiment 2) to validate that the difference between the consonants in /aba/ (token A1) and /ada/ (token A9) would elicit an MMN. This is a prerequisite for the elicitation of a McGurk-MMN, because if no auditory-only MMN is elicited by an actual difference between the consonants, no

McGurk-MMN is expected either. Furthermore, the auditory-evoked MMN also served as a reference to estimate the time at which an illusory sound change induced by either visual speech or text information would penetrate the auditory system."

Experiment 2:
1) More EOG correction information is needed.

Response: We added more information about the EOG correction: "After EOG correction (by applying the Gratton *et al.* (1983) algorithm in which ocular artifacts were corrected by subtracting the EOG channels, multiplied by a channel-dependent correction factor from the EEG channels) epochs with an amplitude change exceeding ±120 μV at any EEG channel were rejected (rejection rate for standard and deviant was 12.7% and 13.2%, respectively)"

2) Please add statistics to the results section.

Response: We added the following: "The mean activity was calculated per phase and separately entered in a repeated measures ANOVA with the within-subject variable Electrode (PO3, POZ, PO4, O1, Oz, O2 for the 90-150 ms phase and Fz, FC1, FC2, C3, Cz, C4, CP1, CP2, Pz for the 180-350 ms and 420-650 ms phases). For all three phases the mean activity was more negative than zero ($F_{1,21} = 7.42$, $P = .01$, $\eta_p^2 = .26$; $F_{1,21} = 56.11$, $P = 2.3 \times 10^{-7}$, $\eta_p^2 = .73$; $F_{1,21} = 15.56$, $P = .0007$, $\eta_p^2 = .43$, respectively), independently of Electrode (all p-values < .08)."

3) There is little discussion about the timing of the MMN. In the introduction, the MMN is described as being around 150-250ms after deviant onset, but all the MMNs listed in the paper are much later. Can the authors comment on this?

Response: Not all MMNs were late: the auditory-only MMN did not start as late as the reviewer suggests (i.e., ~180 ms). However, the McGurk MMN for visual speech did indeed started later (~280 ms). We now discuss this finding at the end of the discussion of Experiment 3: "The McGurk-MMN in our study consisted of three phases. This is in line with Experiment 2 in which we also found multiple phases in the MMN, though these acoustically-evoked MMN started earlier (~180 ms). As MMN latency is longer with decreasing stimulus deviation (Näätänen et al., 2007), a possible explanation for the relative late onset of the McGurk MMN is that the visually induced bias of the ambiguous token resulted in a smaller perceptual difference between standard and deviant when compared to the auditory-only condition."

Experiment 3:
1) The videos were shortened. Can the authors elaborate?

Response: We added the rationale for the shortened videos: "The magnitude of the MMN is sensitive to the interstimulus interval (ISI), with larger MMNs for shorter ISIs (Näätänen *et al.*, 2007). In order to improve the conditions to obtain a robust MMN, we therefore stripped a few frames from the video in which there was no essential movement in order to keep the ISI as short as possible."

2) When describing the EEG analysis, was the V-only deviant waveform subtracted from the AV deviant waveform? This step of the analysis is unclear.

Response: No. This is not what we did and it is not described as such in the manuscript. We subtracted the V-only *difference wave* from the AV *difference wave*. In order to prevent any confusion we have rewritten the specific sentence: To suppress ERP activity evoked by the visual change, the difference waveform (deviant – standard) of the V-only condition was subtracted from the difference waveform (deviant – standard) of the AV condition."

3) The method of subtracting V-only from AV seems a little strange. The difference waveform would include deviant activity AND multisensory information. From the subtraction waveforms in Figure 6, it seems that the difference between V-only standard and V-only deviant was small and the same was true for the AV condition. Therefore, the MMN seems to be due to larger negativity in the AV condition, that was present in both standard and deviant waveforms. Can the authors provide a better rationale for their method, when the A-only condition used the typical deviant-standard method? Might the 'early MMN' be due to the effect of A+V?

Response: In the AV condition the standard and deviant differ only with respect to the visual stimulus (aba vs. ada), the sound does not change. The AV difference wave (AV deviant – AV standard) might therefore be composed of 1) auditory-linked MMN due to an (illusory) change in the auditory percept and 2) visual-linked MMN due to the actual visual deviancy. Note that we do not know the relative contribution of factor 1 and 2 to the AV difference wave. The AV difference wave

itself therefore is not suitable to assess the detection of the illusory sound change as reflected in the MMN. So we applied a procedure as suggested and applied by Saint-Amour, De Sanctis, Molholm, Ritter, and Foxe (2007) who wrote a paper about the McGurk MMN. This entails subtracting the V-only difference wave from the AV difference wave to correct for the V-only MMN contribution to the AV MMN. This procedure we describe in the manuscript. We obtain the (McGurk) MMN when the difference wave for the AV condition is more negative than for the V-only condition. This is what is found in Experiment 3 but not in Experiment 4.

4) Why do the electrodes included in the statistics differ depending on the time-points used? How were these electrodes selected?

Response: The selection of electrodes was based on the spatiotemporal analysis by means of the running t-tests. For a particular cluster we determined the scalp distribution and selected the electrodes in the area in which the maximal amplitudes were located.

Experiment 4:

1) Did the text still differ in SOA from the audio?

Response: No. In the description of Experiment 4 it is stated: “The text was synchronized with sound onset (so ~140 ms before the onset of the critical consonant).”

2) The analytical and statistical comments from those listed for Experiment 3 are also relevant here

Response: we already responded to this particular comment earlier (comments 3 and 4).

General Discussion:

1) The presence of a P3 in the AV-text condition does not constitute a null result. Please clarify.

Response: The reviewer is correct, but we restricted our interpretation to a specific time-domain in which we had clear predictions about when an MMN, and its pre-attentive perceptual connotation, was to occur. Our expectation was to find the (McGurk) MMN. The experiment produced a null result as we did not find the MMN. Although we found an (unexpected) P3, our hypothesis was clearly about the MMN. So regarding our hypothesis we found null-result.

2) There is a small problem of generalising across these studies as each experiment contained new participants. It is possible that the AV-text participants generated smaller ERPs overall, which could account for these results. Was there any overlap in participant population?

Response: There was no overlap in participants across the experiments. We have no reason to assume that the participants in the AV-text experiment generated smaller ERPs because the participants were drawn from the same (rather homogenous) healthy population as for the visual speech MMN experiment. But for the sake of the argument, what if for some unexplained reason (thicker skull?), the ERPs were smaller in Experiment 4. Could that explain the difference in results between Experiment 3 and 4? We believe that would not be the case because the ERPs for both standard and deviant would then be equally smaller and therefore the difference wave would not be affected. But again, there is no reason to assume that the participants in Experiment 4 produced smaller ERPs.

Figures:

1) Figure 1 – what is the important difference we are supposed to see here?

Response: Figure 2 provides a visual presentation of how the visual and auditory stimuli progress over time. For demonstration we included 3 spectrograms of auditory stimuli: A1 (clear /aba/), A5 (/a?a/) and A9 (clear /ada/). We highlighted the formant transition because the formant transition of F2 was manipulated to create the auditory continuum. We added this information in the figure caption.

2) Figure 2 – the legend needs to be explained.

Response: We now explain the legend in the figure caption.

3) The waveforms are very small – can these be enlarged?

Response: we now made the waveforms more visible.

4) Can the significant parts of the waveforms be highlighted?

Response: We now do.

Reviewer 3
MAIN CRITIQUE

The short title of the manuscript is "No McGurk MMN text-sound integration". However, this conclusion is possibly an artifact caused by behavioral task selection. Specifically, the tasks given to the subjects were not matched between the Experiment 1 (behavioral) and Experiment 4 (text AV). In Experiment 1, the subjects were given a task that required them to integrate the auditory and visual stimuli. In contrast, in Experiment 4, the task was to attend to the visual stimuli and push button for occasional target stimuli (uppercase), which did not require integrating the auditory and visual text. As the authors correctly note on p. 20, "the magnitude of the MMN is dependent on the perceived difference between the standard and the deviant (Pakarinen et al., 2007)". Thus, it is to be expected that Experiment 1 found an effect for text, but Experiment 4 did not. Therefore, it would be possible to argue that the present manuscript does not have ERP data to investigate what actually happens during AV integration of letters.

Lipreading speech (McGurk-type) designs do not necessarily involve a task requiring AV integration, as the percepts are changed merely by attending to the stimuli. Thus, there is no need for an overt task that requires AV integration. It should also be noted that the magnitude of the perceptual McGurk illusion seems to be correlated with the related brain responses (Benoit et al., 2010).

Along the same lines, several previous publications have reported MMN-type responses using behavioral tasks that do not require audiovisual integration of text and speech (e.g., Froyen et al., 2008; Andres et al., 2011; Mittag et al., 2011). Such findings would seem to suggest that AV integration of text is also automatic. This makes the present negative result particularly novel and interesting.

To clarify the role of the tasks, it might be worthwhile to collect new data similar to Experiment 4 but to employ a task that requires the subjects to integrate the visual and auditory text. However, it would also be possible to interpret the present results in the light of a recently published report suggesting that audiovisual attention boosts ERPs associated with letter-sound integration (Mittag et al., 2013). To clarify, in my opinion the present results are already interesting enough to merit publication, without addition of new data.

To clarify the above, it would be useful to add in Discussion a brief review of the role of tasks in previous literature and how these may have influenced the results. The currently offered speculations on why the ERP results disagree with previous studies (Discussion pp. 20-22) seem somewhat of a stretch, but interesting and novel nevertheless.

Response: The reviewer suggests that the difference in results between the behavioral experiment (Exp 1) and the AV text MMN experiment (Exp 4) is due to the use of different tasks. He/she argues that in the behavioral experiment participants were ‘forced’ to integrate text and speech, but not in the MMN experiment, and this then might impact the results.

We are afraid that there is a misunderstanding because participants in Exp 1 were asked to categorize the auditory tokens **while ignoring the visual stimuli**. The visual stimuli were thus completely task-irrelevant , although they had to be watched. Active attention to both modalities was thus not necessary to obtain a shift in auditory perception.

We further want to stress that the behavioral task in Experiment 1 provided us with a behavioral correlate of AV integration. This task is quite standard and has been used many times in other cross-modal studies. The reviewer argues that in an MMN paradigm no behavioral task is necessary because AV integration is automatic. We completely agree with the reviewer here. That is why there was no behavioral task in both MMN experiments (as customary in MMN paradigms). The reviewer further recommend that we could redo Experiment 4 but now with a task that requires the subjects to integrate the visual and auditory text. However, we do not follow this logic because the reviewer earlier came to the conclusion that AV integration is automatic and therefore a behavioral

task is not necessary. Furthermore, such a task (involving overt attention to the standard and deviant) could introduce an N2b component which might obscure the McGurk MMN.

MINOR POINTS

It was unclear if the deviant probability (apparently 20%) was the same across all ERP studies. Please clarify.

Response: the deviant probability was identical across the MNN studies and is now explicitly stated accordingly.

For Experiment 3 the manuscript states the following: "The novel finding is that the current results exclude the possibility that the McGurk-MMN is induced by a change in audiovisual congruence of the deviant. Rather, it is more likely that an illusory change in sound identity evokes a McGurk-MMN." It could be interesting to relate this conclusion to the results in (Sams et al., 1991) where it would appear that stimulus probability, and not AV congruency, was the factor driving the McGurk-type difference response.

Response: In other studies aimed at eliciting the McGurk MMN by visual speech the standard was AV congruent and the deviant was AV incongruent. The McGurk MMN in these studies may be composed of 1) an MMN evoked by a detection of an (illusory) auditory deviancy that was induced by the McGurk effect in the deviant and 2) a difference between the standard ERP and the deviant ERP just because the standard is congruent and the deviant is incongruent, both producing different ERPs (see also Stekelenburg & Vroomen (2007), JoCN, 17, 1964-1973). We argue that in our study – by using the ambiguous speech token halfway between the /ada/ and /aba/ – the AV incongruency is virtually identical for both standard and deviant, thereby basically eliminating the contribution of the congruency effect (2) to the McGurk MMN. Stimulus probability affects component 1) but not component 2). However, we do not see how discussing the paper of Sams would help to interpret our results because Sams ran two AV conditions with the same probabilities (standard 84%, deviant 16%): a) AV congruent standard and AV incongruent deviant; b) AV incongruent standard and AV congruent deviant. Both conditions produced similar MMNs. The contribution of AV congruency to the MMN is identical in both conditions. So, in our opinion AV congruency could still drive the MMN in the Sams study.

Related to this, in Discussion, would it make sense to separate probability (effective ISI, separately for standards and deviants) vs. AV congruency effects?

Response: Yes, therefore we added the following: "A future study might further investigate the contribution of AV congruency to the McGurk-MMN by manipulating stimulus probability. An 80% standard 20% deviant condition may be compared to a 50%/50% condition. No MMN is to be expected for the 50%/50% condition if the McGurk-MMN is the result of an illusory change in sound identity. However, if a MMN-like response would be found for the 50%/50% condition, this can be ascribed to AV congruency."

Judging by the pre-stimulus baselines in Figures 3, 6, and 7, the data in Experiments 3-4 are quite noisy, more so than for Experiment 2. Can you suggest any reason why this might be? What were the numbers of accepted trials? For ERPs, please report the numbers of accepted trials for each experiment, and within each experiment, for each averaging class.

Response: This is most likely the result of the difference in the number of subtractions. For Experiment 2 there was 1 subtraction (deviant – standard). For Experiments 3 and 4 there were 2 subtractions AV (deviant – standard) – V (deviant – standard). More noise is to be expected with increasing subtractions.

We now included the rejection rates.

Figure 5 shows scalp topography results for both Experiment 3 (lipread speech) and Experiment 4 (text stimuli). The figure legend lists these as for the "AV-V difference wave". Is this merely the AV-V subtraction, and if so, for standards or deviants? Or was there first the subtraction AV-V, and then the subtraction deviant-standard? The caption would benefit from clarification.

Response: We now clarified the subtraction in the legend and the caption.

For Experiment 4 it was unclear if the target ("catch") trials were averaged separately or together with the non-target trials. These should be averaged separately. Please clarify.

Response: In the original manuscript we state that measurements, and analyses were identical to Experiment 3, but now have added that the catch trials were not included: “Figure 7 shows the ERPs for the standard and deviant (only non-catch trials were included) with their corresponding difference waves for the AV and V condition.”

Discussing the results of Andres et al (2011) on pp. 21-22 the authors write: "It may, however, be questioned whether the neural generators underlying the change detection process in the auditory cortex were actually affected by written text because the difference between the incongruent and congruent MMN was found at a parietal electrode (Pz) and not at the frontal sites where both the MMN of the congruent and incongruent AV stimuli had their topographical maximum. It is therefore uncertain whether the intersensory effect at Pz can actually be traced back to a difference in strength of the neural generators underling the MMN." The MMN has generators not only in the temporal auditory cortices but also in the frontal and parietal cortex (e.g., (Alho, 1995; Rinne et al., 2000; Opitz et al., 2002; Molholm et al., 2005; Restuccia et al., 2005)). Thus, particularly since the present manuscript does not use source localization, and because the spatial relationship between scalp EEG and brain generators is complex, this conclusion seems premature. Moreover, if I am reading Figure 4 correctly, it seems that the MMN topography in present Experiment 2 was maximal at Cz/Pz, not at Fz.

Response: We agree that our reasoning here could be made stronger. We changed this paragraph accordingly: “The difference between the incongruent and congruent MMN, however, was found at a parietal electrode (Pz) and not at the frontal sites where both the MMN of the congruent and incongruent AV stimuli reached their topographical maximum. Manipulating AV congruency may have activated an additional neural generator in one of both conditions that projected to the posterior electrodes on the scalp. It might therefore be questioned whether the difference between the incongruent and congruent MMN reflects a difference in the change detection process in the auditory cortex or rather an AV congruency effect. We conjecture that the results of the above discussed MMN studies could also be explained by letter-sound congruency, and do not provide conclusive evidence that written text actually changes pre-attentive auditory deviancy detection.”

In the above excerpt from Discussion, note the typo ("underling").

Response: fixed.

In future analyses, would it be possible to take the between-subjects differences in the degree of perceptual effects taken into account? See for example (Benoit et al., 2010).

Response: If the reviewer alludes to correlating the behavioral scores (of the McGurk effect) to the MMN scores, we would agree that this would add to our understanding of the link between behavioral and neural level. Unfortunately, in the current study different subjects participated in different experiments, which prevents us from doing this analysis on the current dataset.

In Discussion, it could be useful to separate interaction (A+V-AV) vs MMN paradigms when discussing early vs. late congruency effects. Perhaps it could also be useful to note that cross-modal activations and

interactions between auditory and visual cortices in the human brain seem ubiquitous, as the supratemporal auditory cortex may be activated by visual stimuli even without any auditory associations (checkerboards) starting at about 75 ms after stimulus onset, followed by audiovisual interactions starting at about 80 ms (Raij et al., 2010).

> Response: The distinction between A+V-AV vs. MMN is hard to make because some non-MMN studies we discuss, used the additive model approach (A+V-AV) while others do not, but manipulate only AV congruency. To not complicate matters unnecessarily we opt for sticking to our original text in which we already separated MMN studies from studies reporting AV congruency effects.

No source analysis (localizing the cortical generators of the scalp EEG responses) was used. The spatial relations between scalp EEG signals and their intracranial generators is very complex, especially for sulcal sources where the scalp maxima can be very far from the generators. Thus, it is unclear where in the brain the observed ERPs were generated, which hampers comparisons against studies that have used source analysis techniques. The sensor-space scalp topography maps are shown, but these are not very informative. I do not think that in the present study the lack of source analysis is a problem severe enough to prevent publication, but including it would have increased the appeal.

> Response: We agree that inferring sources from scalp is complex (especially for EEG) and scalp topographies are not very useful in that respect. On the other hand, we know from numerous MMN studies that the MMN is maximal at the frontal-central sites. If an occipital maximum is found this would raise some questions about the identity of this particular component of the difference wave. However, source analysis in our study is not very useful because we found no MMN in Experiment 4. Furthermore, 32 electrodes is rather low to acquire accurate inverse solutions.

Figure 6 legend should state that the results are for Experiment 3 (for example, see Figure 3 legend).

> Response: Fixed.

Figure 7 legend should state that the results are for Experiment 4 (for example, see Figure 3 legend).

> Response: Fixed.

2nd Editorial Decision 15 January 2018

Dear Dr. Stekelenburg,

Your revised manuscript was re-evaluated by two of the original external reviewers and ourselves. We are pleased to inform you that we expect that it will be acceptable for publication in EJN following one more round of what are mostly minor revisions.

If you are able to respond fully to the points raised, we shall be pleased to receive a revision of your paper within 30 days.

Thank you for submitting your work to EJN.

Kind regards,

John Foxe & Paul Bolam
co-Editors in Chief, EJN

Reviews:

Reviewer: 2 (Sarah Haigh, University of Pittsburgh, USA)

EJN European Journal of Neuroscience FENS Federation of European Neuroscience Societies

Comments to the Author
I reviewed this paper on its previous submission. The authors answered my questions, and I do not have any further comments. This revision is much clearer. Thank you.

Reviewer: 3 (Tommi Raij, Northwestern University, USA)

Comments to the Author
The authors were mainly responsive to the reviewer critiques. As a result, the manuscript has improved and is close to publication quality. However, there are still some points - most of them minor - that would benefit from fixing before acceptance.

Title
The new title has a problem. "Only visual speech induces the McGurk visual mismatch negativity (MMN)" indicates that letters do not induce the McGurk mismatch negativity. The problem is, the McGurk phenomenon, by definition, exists for visual speech only, so it would be stating the obvious that it does not exist for other types of stimuli. Perhaps delete the word "McGurk" from the title?

Abstract
The original manuscript seemed to occasionally confuse the lack of MMN with a lack of any responses related to audiovisual integration of letters, which was a problem noted by more than one reviewer. In the present revision, this confusion is slightly alleviated but at times continues. The manuscript should be revised to clearly state, starting already in the Abstract, that previous research has found compelling evidence that text does influence the neuronal processing of auditory letters (for intracranial and non- invasive evidence see, e.g., (Chan, et al. 2014; Raij, et al. 2000; van Atteveldt, et al. 2004)). Further, the manuscript, including the Abstract, should clearly state that the present study also found an ERP component consistent with that text influences the processing of auditory letters (Figure 7 "P3b"). In a sense, whether this response fits more nicely in the MMN or P3b locker is not that all-important - brain activity is brain activity, no matter what labels we give to the different bumps.
To this tune, the conclusion in the Abstract "These results demonstrate that text has much weaker effects on sound processing than visual speech does, possibly because text has different biological roots than visual speech." would benefit from toning down. A conclusion that would more accurately reflect the present results would be that (during a task that does not require audiovisual integration of letters (?)) there was no typical MMN response, although there was another evoked response component consistent with that text influences sound processing.

MAIN CRITIQUE

Author response in R1:
"The reviewer suggests that the difference in results between the behavioral experiment (Exp 1) and the AV text MMN experiment (Exp 4) is due to the use of different tasks. He/she argues that in the behavioral experiment participants were ‘forced’ to integrate text and speech, but not in the MMN experiment, and this then might impact the results.

We are afraid that there is a misunderstanding because participants in Exp 1 were asked to categorize the auditory tokens while ignoring the visual stimuli. The visual stimuli were thus completely task-irrelevant, although they had to be watched. Active attention to both modalities was thus not necessary to obtain a shift in auditory perception.

We further want to stress that the behavioral task in Experiment 1 provided us with a behavioral correlate of AV integration. This task is quite standard and has been used many times in other cross-modal studies. The reviewer argues that in an MMN paradigm no behavioral task is necessary because AV integration is automatic. We completely agree with the reviewer here. That is why there was no behavioral task in both MMN experiments (as customary in MMN paradigms). The reviewer further recommend that we could redo Experiment 4 but now with a task that requires the subjects to integrate the visual and auditory text. However, we do not follow this logic because the reviewer earlier came to the conclusion that AV integration is automatic and therefore a behavioral task is not necessary. Furthermore, such a task (involving overt attention to the standard and deviant) could introduce an N2b component which might obscure the McGurk MMN."

EJN European Journal of Neuroscience FENS Federation of European Neuroscience Societies

Reviewer comments for R1:

This response did not quite adequately address the problems stated in the critique. Regarding the nature of the tasks and balancing them across the Experiments:
This reviewer has trouble understanding how these seemingly contradictory statements can be true at the same time (quotes from the authors' response to reviewers):
- "participants in Exp 1 were asked to categorize the auditory tokens while ignoring the visual stimuli. The visual stimuli were thus completely task-irrelevant,"
- "the behavioral task in Experiment 1 provided us with a behavioral correlate of AV integration."
Please clarify. If it is the case that the tasks in Experiments 1 versus 4 were not balanced in the requirement to behaviorally integrate the auditory and visual stimuli, then my original critique stands. If it is true that Experiment 1 did not require behavioral AV integration, the text should state this clearly.

Regarding the role of attention, automatic processing, and tasks:
The authors responded that "The reviewer argues that in an MMN paradigm no behavioral task is necessary because AV integration is automatic." Here, they are misquoting/misunderstanding the reviewer. I do not think that AV integration is very automatic. Further, I would take the somewhat controversial position that MMNs are modulated by attention and therefore by task.
First, it is first useful to note that while the categorization [attention is required] vs. [processing is automatic] is often presented as binary, in terms of behavior and brain function, there are plenty of shades in between, and there are several types of attention. The level and type of attention influences ERPs, including those in the MMN latency range, shown by several experimental papers and reviews (Auksztulewicz and Friston 2015; Campbell 2014; Erlbeck, et al. 2014; Sussman, et al. 2014; Woldorff, et al. 1998).

Then, it should be expected that response amplitudes, including those in the MMN latency range, will depend on the degree and type of attention, which is dictated by the task (mainly, if it requires behavioral integration between the text and sound or not). This led to my suggestion to add new data with different attentional demands, or otherwise discuss the role of attention and task in more detail. As long as there are no new data similar to Experiment 4 with a task that requires the subjects to integrate the visual and auditory text, one cannot know if this new experiment would evoke MMN-type responses. This is a point (to be addressed in future studies) that should be mentioned in Discussion. Overall, it should be clearly stated in Discussion that none of the tasks in any of the experiments required the subjects to behaviorally integrate the auditory and visual stimuli (if the authors agree that this was the case).

MINOR POINTS

All minor points were adequately addressed.

References

Auksztulewicz R, Friston K. (2015): Attentional enhancement of auditory mismatch responses: a DCM/MEG study. Cereb Cortex 25(11):4273-83.
Campbell T. (2014): A theory of attentional modulations of the supratemporal generation of the auditory mismatch negativity (MMN). Front Hum Neurosci 2014(8).
Chan A, Dykstra A, Jayaram V, Leonard M, Travis K, Gygi B, Baker J, Eskandar E, Hochberg L, Halgren E and others. (2014): Speech-specific tuning of neurons in human superior temporal gyrus. Cereb Cortex 2679-93(10):2679-93.

Erlbeck H, Kübler A, Kotchoubey B, Veser S. (2014): Task instructions modulate the attentional mode affecting the auditory MMN and the semantic N400. Fron Hum Neurosci 8:654.
Raij T, Uutela K, Hari R. (2000): Audiovisual integration of letters in the human brain. Neuron 28:617-625.
Sussman E, Chen S, Sussman-Fort J, Dinces E. (2014): The five myths of MMN: redefining how to use MMN in basic and clinical research. Brain Topogr 27(4):553-64.

van Atteveldt N, Formisano E, Goebel R, Blomert L. (2004): Integration of letters and speech sounds in the human brain. Neuron 43(2):271-282.
Woldorff M, Hillyard S, Gallen C, Hampson S, Bloom F. (1998): Magnetoencephalographic recordings demonstrate attentional modulation of mismatch-related neural activity in human auditory cortex. Psychophysiology 35:283-292.

Authors' Response 12 February 2018

Title
The new title has a problem. "Only visual speech induces the McGurk visual mismatch negativity (MMN)" indicates that letters do not induce the McGurk mismatch negativity. The problem is, the McGurk phenomenon, by definition, exists for visual speech only, so it would be stating the obvious that it does not exist for other types of stimuli. Perhaps delete the word "McGurk" from the title?

> Response: We acknowledge the problem in the title and followed the recommendation of the reviewer. "Multisensory integration of speech sounds with letters versus visual speech: Only visual speech induces the mismatch negativity (MMN)".

Abstract
The original manuscript seemed to occasionally confuse the lack of MMN with a lack of any responses related to audiovisual integration of letters, which was a problem noted by more than one reviewer. In the present revision, this confusion is slightly alleviated but at times continues. The manuscript should be revised to clearly state, starting already in the Abstract, that previous research has found compelling evidence that text does influence the neuronal processing of auditory letters (for intracranial and non-invasive evidence see, e.g., (Chan, et al. 2014; Raij, et al. 2000; van Atteveldt, et al. 2004)).

> Response: In the abstract we added: "While there is ample evidence for integration of text and auditory speech, there are only a few studies on the orthographic equivalent of the McGurk effect." In the introduction we already cite van Atteveldt, et al. 2004. We now added Raij, et al. 2000: "A MEG study confirmed the involvement of STS in letter-sound integration (Raij *et al.*, 2000)."

Further, the manuscript, including the Abstract, should clearly state that the present study also found an ERP component consistent with that text influences the processing of auditory letters (Figure 7 "P3b"). In a sense, whether this response fits more nicely in the MMN or P3b locker is not that all-important - brain activity is brain activity, no matter what labels we give to the different bumps.

> Response: we added: "We found that only deviant visual speech induced an MMN, but not deviant text, which induced a late P3-like positive potential."

To this tune, the conclusion in the Abstract "These results demonstrate that text has much weaker effects on sound processing than visual speech does, possibly because text has different biological roots than visual speech." would benefit from toning down. A conclusion that would more accurately reflect the present results would be that (during a task that does not require audiovisual integration of letters (?)) there was no typical MMN response, although there was another evoked response component consistent with that text influences sound processing.

> Response: We believe that the conclusion does justice to the findings of our experiments, also considering the role of task/attention we now raise in the discussion.

Regarding the nature of the tasks and balancing them across the Experiments:
This reviewer has trouble understanding how these seemingly contradictory statements can be true at the same time (quotes from the authors' response to reviewers):
- "participants in Exp 1 were asked to categorize the auditory tokens while ignoring the visual stimuli. The visual stimuli were thus completely task-irrelevant,"
- "the behavioral task in Experiment 1 provided us with a behavioral correlate of AV integration."
Please clarify.

> Response: Many, if not most tasks yielding a behavioral correlate of AV integration involve one modality that is not task relevant: e.g. McGurk effect; double flash illusion; ventriloquist effect; Pip-and-pop effect; cross-modal gain with speech in noise, etc. So, there is ample evidence that AV integration can occur in tasks by measuring the effect in one modality induced by the other modality that is task-irrelevant and can be ignored. In our opinion, our statements are therefore not contradictory.

If it is the case that the tasks in Experiments 1 versus 4 were not balanced in the requirement to behaviorally integrate the auditory and visual stimuli, then my original critique stands.
If it is true that Experiment 1 did not require behavioral AV integration, the text should state this clearly.

> Response: We added in the method section that the visual stimulus was not task-relevant. “The task of the participants was to identify the sounds as either /aba/ or /ada/ using two dedicated buttons, while ignoring (but still watching) the visual stimulus.”

Regarding the role of attention, automatic processing, and tasks:
The authors responded that "The reviewer argues that in an MMN paradigm no behavioral task is necessary because AV integration is automatic." Here, they are misquoting/misunderstanding the reviewer. I do not think that AV integration is very automatic. Further, I would take the somewhat controversial position that MMNs are modulated by attention and therefore by task.
First, it is first useful to note that while the categorization [attention is required] vs. [processing is automatic] is often presented as binary, in terms of behavior and brain function, there are plenty of shades in between, and there are several types of attention. The level and type of attention influences ERPs, including those in the MMN latency range, shown by several experimental papers and reviews (Auksztulewicz and Friston 2015; Campbell 2014; Erlbeck, et al. 2014; Sussman, et al. 2014; Woldorff, et al. 1998).
Then, it should be expected that response amplitudes, including those in the MMN latency range, will depend on the degree and type of attention, which is dictated by the task (mainly, if it requires behavioral integration between the text and sound or not). This led to my suggestion to add new data with different attentional demands, or otherwise discuss the role of attention and task in more detail. As long as there are no new data similar to Experiment 4 with a task that requires the subjects to integrate the visual and auditory text, one cannot know if this new experiment would evoke MMN-type responses. This is a point (to be addressed in future studies) that should be mentioned in Discussion.
Overall, it should be clearly stated in Discussion that none of the tasks in any of the experiments required the subjects to behaviorally integrate the auditory and visual stimuli (if the authors agree that this was the case).

> Response: we addressed the issues raised by the reviewer in the discussion:
> “A factor that might account for differences between letter-sound integration in the behavioral task (Experiment 1) and the MMN task (Experiment 4) might relate to differences in attention payed to the letters. In Experiment 1, speech sounds had to be rated while letters were to be ignored, whereas in Experiment 4, speech sounds and letters where both task-irrelevant. What these two Experiments have in common is that the tasks themselves did not require AV integration, and that the letters were always task-irrelevant. It seems therefore doubtful that differences in attention payed to letters would explain differences between the behavioral and neural data, and why this putative effect of attention would solely occur for letters, but not visual speech. A future study, though, might explore whether letter-sound integration at the neural level can be boosted by requiring participants to relate the speech sounds and text to each other (cf. Raij *et al.*, 2000).”
